# Extension of mRNA poly(A) tails and 3′UTRs during neuronal differentiation exhibits variable association with post-transcriptional dynamics

**DOI:** 10.1093/nar/gkad499

**Published:** 2023-06-09

**Authors:** Dylan J Kiltschewskij, Paul F Harrison, Chantel Fitzsimmons, Traude H Beilharz, Murray J Cairns

**Affiliations:** School of Biomedical Sciences and Pharmacy, The University of Newcastle, Callaghan, NSW 2308, Australia; Precision Medicine Research Program, Hunter Medical Research Institute, New Lambton Heights, NSW 2305, Australia; Department of Biochemistry and Molecular Biology, Monash University, Clayton, VIC 3800, Australia; School of Biomedical Sciences and Pharmacy, The University of Newcastle, Callaghan, NSW 2308, Australia; Precision Medicine Research Program, Hunter Medical Research Institute, New Lambton Heights, NSW 2305, Australia; Department of Biochemistry and Molecular Biology, Monash University, Clayton, VIC 3800, Australia; School of Biomedical Sciences and Pharmacy, The University of Newcastle, Callaghan, NSW 2308, Australia; Precision Medicine Research Program, Hunter Medical Research Institute, New Lambton Heights, NSW 2305, Australia

## Abstract

Differentiation of neural progenitor cells into mature neuronal phenotypes relies on extensive temporospatial coordination of mRNA expression to support the development of functional brain circuitry. Cleavage and polyadenylation of mRNA has tremendous regulatory capacity through the alteration of mRNA stability and modulation of microRNA (miRNA) function, however the extent of utilization in neuronal development is currently unclear. Here, we employed poly(A) tail sequencing, mRNA sequencing, ribosome profiling and small RNA sequencing to explore the functional relationship between mRNA abundance, translation, poly(A) tail length, alternative polyadenylation (APA) and miRNA expression in an *in vitro* model of neuronal differentiation. Differential analysis revealed a strong bias towards poly(A) tail and 3′UTR lengthening during differentiation, both of which were positively correlated with changes in mRNA abundance, but not translation. Globally, changes in miRNA expression were predominantly associated with mRNA abundance and translation, however several miRNA–mRNA pairings with potential to regulate poly(A) tail length were identified. Furthermore, 3′UTR lengthening was observed to significantly increase the inclusion of non-conserved miRNA binding sites, potentially enhancing the regulatory capacity of these molecules in mature neuronal cells. Together, our findings suggest poly(A) tail length and APA function as part of a rich post-transcriptional regulatory matrix during neuronal differentiation.

## Graphical Abstract

**Graphical Abstract:**
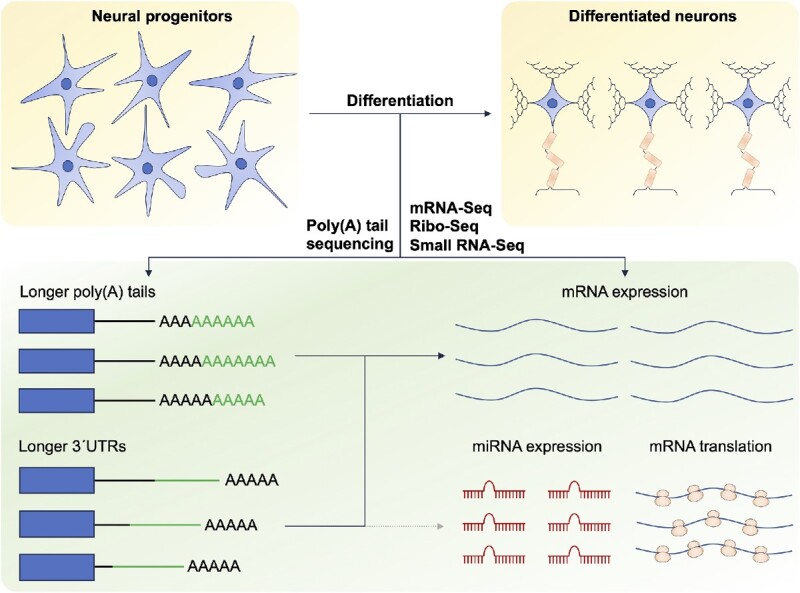

## INTRODUCTION

The generation of neuronal subpopulations with unique morphological and electrophysiological properties is driven by discrete patterns of mRNA expression in response to developmental cues. It is currently well established that activation of neuron-specific mRNA expression is largely mediated by an array of transcription factors, that modify the transcriptome throughout the course of the differentiation process (1–3). In contrast, our knowledge of the post-transcriptional regulatory systems which fine-tune mRNA expression during neuronal differentiation is less comprehensive. Although a vast collection of *cis*- and *trans*-acting mechanisms contribute to mRNA localisation, stability and translational capacity during development and differentiation (4–6), the behaviour and interplay of these mechanisms remains elusive. This is significant, given that dysregulation of post-transcriptional systems has emerged as a common feature amongst several psychiatric disorders associated with disrupted neuronal development, such as schizophrenia, bipolar disorder and autism spectrum disorders (7,8).

One dimension of mRNA biology thought to be pivotal in multiple aspects of post-transcriptional regulation is cleavage and polyadenylation, a near ubiquitous mRNA end processing mechanism intricately coupled with transcriptional termination. Canonically, pre-mRNA cleavage is initiated downstream of conserved polyadenylation site motifs, of which the A[A/U]UAAA motif is most prominent, following which poly(A) polymerase synthesises a poly(A) tail of approximately 50–100nt (9,10). An intact poly(A) tail is critical throughout the life cycle of an mRNA, given this structure is required for mRNA nuclear export (11), confers resistance to degradation (12) and enables association with poly(A) binding proteins for enhancement of translational efficiency (13). Furthermore, erosion of the poly(A) tail by CCR4-NOT and PAN2-PAN3 complexes constitutes the predominant mechanism through which microRNAs (miRNAs) instigate degradation of partially complementary mRNAs in eukaryotic cells (14–16). In neurons, cytoplasmic polyadenylation may also regulate translational competency of mRNAs in close proximity to the synapse (17,18). While these facets of poly(A) tail dynamics suggest a particularly important role in post-transcriptional regulation, global changes in polyadenylation and their association with mRNA dynamics and miRNA expression during neuronal differentiation are yet to be characterised.

Alternative polyadenylation (APA) can also drastically impact post-transcriptional dynamics by modifying utilisation of polyadenylation signals (PAS) (19–23). These changes directly affect 3′UTR length and subsequently impact key regulatory motifs such as AU-rich elements (24), localisation elements (25) and miRNA binding sites (26), all of which contribute to mRNA stability and translational status (27). Interestingly, cell-specific APA profiles have been recently discovered, with proliferative cells generally expressing short 3′UTR APA isoforms (28), whereas complex, terminally differentiated cells such as neurons are often associated with longer 3′UTRs (29). During neuronal differentiation, the functional consequences of APA have recently been described for specific genes such as *Bdnf* (30) and *Rac1* (31), while emerging studies have identified a transition towards long 3′UTR expression during neuronal differentiation (32,33) and brain development (34,35), indicating a particularly important role in this phenotypic transition.

The recent development of high-throughput strategies for poly(A) tail and 3′UTR quantification (36,37) now provide an opportunity to explore these broad regulatory mechanisms on a genome-wide scale. To this end, we employed genome-wide poly(A) tail sequencing (PAT-Seq) (36), mRNA sequencing (mRNA-Seq), ribosome profiling (Ribo-Seq) and small RNA sequencing to gain further insight into the regulation of mRNA cleavage and polyadenylation during neuronal differentiation, and their interplay with mRNA and miRNA dynamics. Our study revealed many genes subjected to poly(A) tail and/or 3′UTR lengthening during neuronal differentiation, both of which were associated with mRNA expression levels. We also identify miRNA–mRNA pairings associated with poly(A) tail length modulation and explore the impact of 3′UTR lengthening on miRNA binding sites. These results suggest lengthening of poly(A) tails and 3′UTRs constitutes an important mechanism of post-transcriptional mRNA regulation associated with neuronal differentiation.

## MATERIALS AND METHODS

### Cell culture

Human SH-SY5Y neuroblastoma cells were grown and sustained in Dulbecco's Modified Eagle's Medium (HyClone, Logan, UT, USA), supplemented with 10% heat-inactivated fetal bovine serum (Bovogen Biologicals, Essendon, VIC, Australia), 2% HEPES (Gibco, Carlsbad, CA, USA) and 1% l-glutamine (Invitrogen, Carlsbad, CA, USA). Cultures were maintained at 37°C in a 95% oxygen, 5% carbon dioxide, 90% humidity atmosphere and passaged regularly by washing with phosphate buffered saline (Gibco) and trypsinisation (0.1% trypsin–EDTA in PBS, Gibco). All cells used in this study were passage 9 at the time of differentiation. All experiments were conducted in biological triplicate.

### Neuronal differentiation

**Figure 1.**
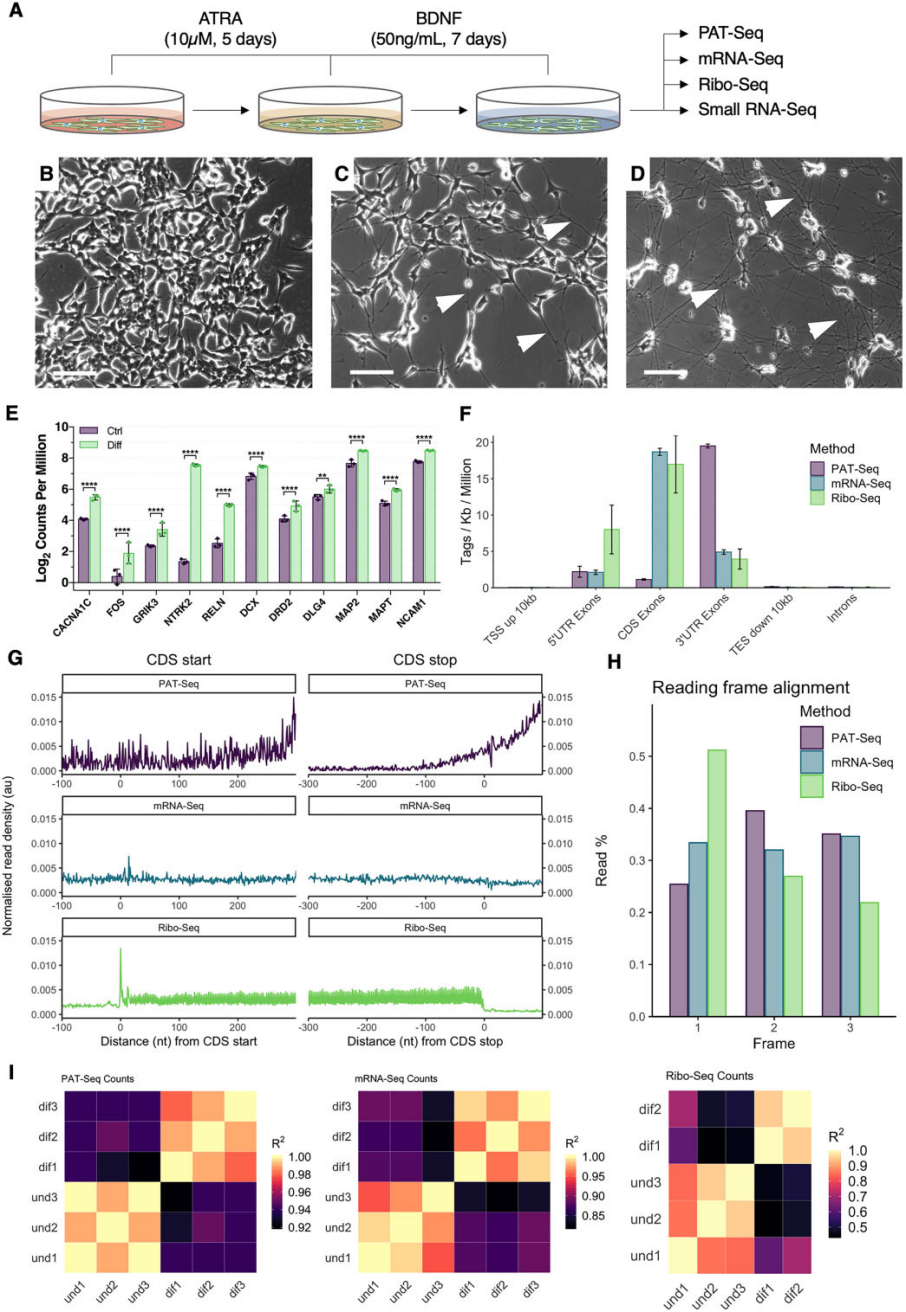
Sequencing of neuronally differentiated SH-SY5Y cells. (**A**) Schematic overview of the neuronal differentiation paradigm and sequencing strategies employed in this study. (B–D) Phase contrast micrographs depicting SH-SY5Y cells cultured under standard conditions (**B**), 5 days after media supplementation with ATRA (**C**), and after ATRA treatment followed by an additional 7 days of serum starvation and BDNF treatment (**D**). Note the extensive development of neurites after differentiation relative to naïve cells (white arrowheads). Scale bar = 100 μm. (**E**) Expression of known neuronal marker genes in control (purple) and differentiated (green) cells, as quantified via mRNA sequencing. All genes were subjected to statistically significant upregulation (Benjamini–Hochberg *FDR* < 0.05), with *CACNA1C*, *FOS*, *NTRK2* and *RELN* additionally subjected to >2-fold increased expression relative to naïve cells. Data presented as log_2_ counts-per-million (CPM) ± SD. **FDR < 0.01, ****FDR < 0.0001. (**F**) Genomic distribution of uniquely aligned reads for mRNA-Seq, PAT-Seq and Ribo-Seq libraries. Data presented as assigned tags per kilobase per million assigned tags ± SD. (**G**) Metagene analysis of read density around CDS start and stop codons. Note that PAT-Seq libraries exhibit increased read density in a 5′ to 3′ direction, particularly around CDS stop codons. (**H**) Sub-codon phasing of all three libraries. Note that Ribo-Seq reads exhibit bias for the first reading frame as expected for ribosome-protected fragments. (**I**) Correlation of gene-level read-count profiles amongst biological replicate samples. One replicate from the differentiated group was excluded from the Ribo-Seq experiment due to poor data quality.

Neuronal differentiation was induced via sequential treatment with *all-*trans retinoic acid (ATRA, Sigma-Aldrich, St. Louis, MO, USA) and brain-derived neurotrophic factor (BDNF, Life Technologies, Carlsbad, CA, USA), as previously described (38). One day prior to differentiation (day -1), cells were seeded into T75 culture flasks at a density of 25 000 cells/cm^2^ in standard culture medium. The next day (day 0), standard medium was replaced with ATRA-supplemented medium (10μM) and cells were protected from light exposure, with ATRA medium replaced on day 2. On day 5, ATRA medium was aspirated, cells were washed 3 times with standard culture medium, then returned to standard culturing conditions in BDNF-supplemented (50ng/mL), serum-free medium. BDNF medium was replaced on days 7, 9 and 11, after which the methods were continued as described on day 12. Successful neuronal differentiation was established by examining neurite outgrowth during ATRA/BDNF treatment and analysing the expression of neuronal marker genes (see Figure 1B–E). Differentiated cells were compared to untreated control samples cultured under standard conditions.

### PAT-seq library preparation

Total RNA was extracted from all samples by harvesting cells in 1mL TRIzol reagent (ThermoFisher, Waltham, MA, USA), as per the manufacturer's instructions. Poly(A) tail sequencing (PAT-Seq) was conducted using 1μg of high-quality RNA (RNA integrity number = 10) as previously described (36). Briefly, biotinylated ePAT primers (5′-Bio-CAGACGTGTGCTCTTCCGATCT_(18)_-3′ ; 100μM) were annealed to polyadenylated mRNAs and 3′ ends were extended using 5 units (U) of the Klenow fragment of DNA polymerase I (NEB, Ipswich, MA, USA). RNAs were then subjected to limited RNase T_1_ digestion (10U, Roche, Basel, Switzerland), with parameters optimised to produce ∼250nt fragments, after which 3′ fragments were collected with streptavidin beads (NEB). Phosphorylation of 5′-ends was next performed with poly nucleotide kinase (0.5U, NEB), prior to ligation of 5′ splinted linkers, reverse transcription, and size selection (120–400 bp) via 6% denaturing urea-polyacrylamide gel. To amplify all samples, 10μl of cDNA was subjected to 16 PCR cycles, with 2μl of ScriptSeq indexed reverse primers (50μM, Epicentre, Madison, WI, USA) included for sample indexing compatible with Illumina (San Diego, CA, USA) sequencing platforms. PAT-Seq libraries were subsequently normalised, pooled and sequenced (151 single end cycles) on a single lane of an Illumina HiSeq 1500.

### Quantification of poly(A) tail length and APA events

PAT-Seq data were analysed using the *tail tools* (v.0.41) *R* package (36). Firstly, adapter sequences and poly(A) tails were trimmed from fastq-formatted reads. Poly(A) tails were identified by calling a run of consecutive ‘A’ residues extending to the 3′ end, or the adapter sequence, with an error rate of 1 in 5 bases and Phred33 quality score > 10 allowed. Reads were then aligned to the reference genome (hg19, UCSC) using *Bowtie2* (39) and deemed polyadenylated if a non-templated run of ≥ 4 consecutive ‘A’ residues was detected. For genes with ≥ 10 poly(A) reads, average tail length was calculated. Differential analysis of tail length between experimental conditions was determined using the *limma* (40) voom statistical pipeline, accounting for depth-associated variation in tail length estimations. Benjamini-Hochberg false discovery rate (*FDR_BH_*) < 0.1 and absolute change in tail length > 10 ‘A’ residues were considered significant.

APA events were identified with the *DEXSeq* (v1.38.0) *R* package (41). Poly(A) reads overlapping known MACE-supported 2–30nt APA sites curated from APADB (v2) (21) were counted using the *dexseq_count.py* script. Counts were then converted into a *DEXSeq* dataset, after which library size factors, dispersions and APA fold changes were estimated. Sites with ≥2 normalised counts across all samples were retained, after which differential APA site usage between control and differentiated cells was determined via likelihood ratio test. Relative expression difference (RED) between the two most abundant APA sites (i.e. proximal and distal) across both conditions was then determined as follows:


\begin{equation*}{\mathrm{RED\ }} = {\mathrm{\ lo}}{{\mathrm{g}}}_2\left( {\frac{{{\mathrm{Differentiate}}{{\mathrm{d}}}_{dist}}}{{{\mathrm{Contro}}{{\mathrm{l}}}_{dist}}}} \right) - \ {\log }_2\left( {\frac{{{\mathrm{Differentiate}}{{\mathrm{d}}}_{prox}}}{{{\mathrm{Contro}}{{\mathrm{l}}}_{prox}}}} \right)\ \end{equation*}

Significant lengthening events required RED > 1, coupled with significantly increased (*FDR_BH_* < 0.1) expression of the distal isoform and/or a significant decrease of the proximal isoform. Conversely, significant shortening events required a RED < –1, with a significant decrease in expression of the distal isoform and/or a significant increase of the proximal isoform. APA events were validated via qPCR utilising primers designed to amplify the entire gene, or the long 3′UTR isoform only (see Supplementary Methods for further details, and Supplementary Table S1 for primer sequences).

### mRNA sequencing, ribosome profiling and small RNA sequencing

mRNA library preparation was conducted using the Illumina TruSeq Stranded mRNA Library Preparation Kit, according to the manufacturer's instructions. Briefly, 1μg of total RNA was subjected to poly(A) selection via oligo-DT beads, heat fragmentation (94°C, 4 min), reverse transcription, adapter ligation and amplification via 15 PCR cycles. Libraries were subjected to 151 cycles of single end sequencing using the Illumina NextSeq500 platform.

Ribosome profiling (Ribo-Seq) was performed via the Illumina TruSeq Ribo Profile Kit (H/M/R), with minor amendments. Translational activity was firstly suspended by incubating cells in warm culture medium supplemented with 0.1mg/ml cycloheximide (CHX) for 1 min. Cells were harvested on ice with 800μl mammalian lysis buffer (containing 0.1mg/ml CHX and 25U Turbo DNase I (ThermoFisher)), and ribosome foot-printing was conducted by adding 150U RNase I (Invitrogen) to 300μl clarified lysate for 45 min. Library preparation was conducted per the manufacturer's instructions, with libraries amplified via 12 PCR cycles and subjected to 101 cycles of single end sequencing via a NovaSeq6000 Instrument.

For small RNA sequencing, libraries were produced from 1μg of total RNA using the Illumina TruSeq Small RNA Library Preparation Kit, according to the manufacturer's instructions. Firstly, adapters were ligated to RNAs, prior to reverse transcription and amplification via 11 PCR cycles. Samples were pooled in equimolar ratios and volumes, and small RNAs between ∼20–33nt were purified via 6% native PAGE. The resultant library was subjected to 76 cycles of single end sequencing using an Illumina NextSeq500. See Supplementary Methods for further details.

### Processing of sequencing data

Raw sequencing files were demultiplexed using *bcl2fastq* (Illumina), lane data for each sample were merged and data quality was assessed with *FastQC* (v0.11.5). Low quality 3′ bases (phred33 score < 28) were trimmed and additional quality control measures were employed via *Cutadapt* (v1.14) (42). For mRNA data, libraries were aligned to the reference genome (hg19, UCSC) with *HISAT2* (v.2.0.2) (43) and reads aligning to features were counted with *HTSeq* (v.0.9.1) (44). For Ribo-Seq libraries, sequences aligning to short, abundant noncoding RNAs were identified and discarded using *Bowtie2* (v2.2.6) (39), prior to genome alignment and read-counting with *Tophat2* (v2.2.1) (45) and *HTSeq*, respectively. All mRNA-based libraries were subjected to post-alignment quality control using the *RSeQC* (v2.6.6) (46) and *Plastid* (v0.5.1) (47) packages.

For small RNA data, libraries were firstly aligned to human precursor and mature tRNAs curated from gtRNAdb (v2.0) (48) using *Bowtie2*, for identification of tRNA-derived small RNAs. Unaligned reads were mapped to the NCBI GRCh38 reference genome using *Bowtie2*. This reference genome assembly was selected to ensure compatibility with the miRBase (release 22) (49) mature miRNA annotation file during read-counting via *HTSeq*. See Supplementary Methods for further details.

### Differential expression analysis

Data normalisation and differential expression were conducted with *edgeR* (v3.8) (50). Raw counts for each sample were merged into a single matrix and normalised to sequencing depth (counts-per-million; CPM). Genes and miRNAs with consistently low counts were then removed by employing a minimum CPM threshold, ensuring genes/miRNAs with < 5 raw counts in the smallest library were excluded. Sample clustering and biological variation were assessed by visually inspecting multidimensional scaling (MDS) and biological coefficient of variation (BCV) plots (Supplementary Figures S1, S2). After calculating normalisation factors (TMM method) and estimating dispersion, differential expression between treated and control samples was determined via pairwise exact test, with *FDR_BH_* < 0.05 and absolute log_2_ fold change (log_2_FC) > 1 considered significant. Differential translational efficiency was determined via *RiboDiff* (v.0.2.1) (51). See Supplementary Methods for further details.

### Gene ontology analysis

Gene ontology enrichment was analysed with the *Toppgene* functional enrichment web suite (52), with *FDR_BH_* < 0.05 considered significant.

## RESULTS

### Sequencing of neuronally differentiated SH-SY5Y cells

To investigate 3′UTR dynamics associated with neuronal differentiation, poly(A)-tail sequencing (PAT-Seq), mRNA sequencing (mRNA-Seq) and ribosome profiling (Ribo-Seq) were conducted on SH-SY5Y cells sequentially treated with all-trans retinoic acid (ATRA) and brain-derived neurotrophic factor (BDNF), and untreated controls (Figure 1A). Successful neuronal differentiation was confirmed by examining neurite outgrowth, reduced proliferation, and the acquisition of a polar cellular morphology, which was later supported by the observed upregulation of known neuronal marker genes via mRNA-Seq (Figure 1B–E).

The PAT-Seq methodology produced an average of 6.07 × 10^6^ (±2.83 × 10^6^) polyadenylated (≥ 4nt) reads per sample, which preferentially aligned to 3′UTRs, consistent with a library preparation that constrains reads to the last ∼200 bases of the 3′UTR and poly(A) tail (Figure 1F,G, Supplementary Table S2). mRNA-Seq and Ribo-Seq respectively produced an average of 41.73 × 10^6^ (± 21.55 × 10^6^) and 54.20 × 10^6^ (± 40.20 × 10^6^) aligned reads per sample, which predominantly mapped to CDS exons as anticipated (Figure 1F, G, Supplementary Table S2). Across CDS start and stop codons, mRNA-Seq reads exhibited largely uniform read density characteristic of random fragmentation, whereas Ribo-Seq demonstrated strong peaks and troughs indicative of translation initiation and termination, respectively (Figure 1G). For Ribo-Seq libraries, sub-codon phasing additionally revealed an alignment preference for the first reading frame, indicative of ribosomal triplet periodicity (Figure 1H). Finally, comparison of gene-level read-counts revealed strong correlation (*R*^2^ ≥ 0.84) between biological replicates for all three library preparation methods, except for replicate ‘dif3’ in the Ribo-Seq analysis, which was excluded herein (Figure 1I).

### Poly(A) tail lengthening during neuronal differentiation

**Figure 2.**
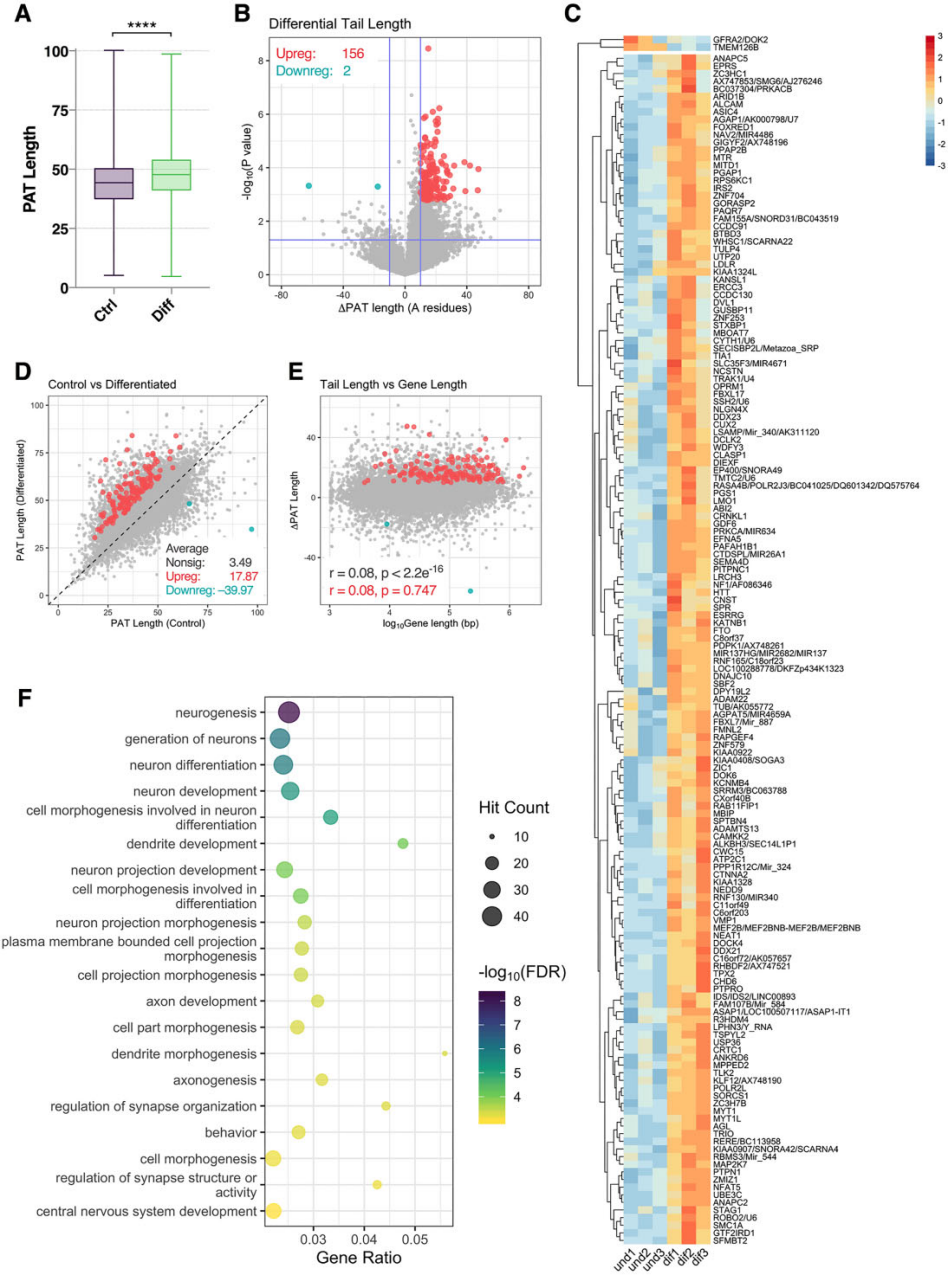
Extension of mRNA poly(A) tails during neuronal differentiation. (**A**) Distribution of poly(A) tail lengths in control and differentiated cells. Groups were compared via Kolmogorov–Smirnov test (****P < 0.0001). (**B**) Volcano plot depicting gene-wise changes in poly(A) tail length versus –log_10_*P*-value. Genes with significantly increased or decreased tail length are marked in red and blue, respectively, with Benjamini–Hochberg *FDR* <0.1 and absolute change in tail length >10 A residues considered significant. Horizontal blue line represents nominal *P* < 0.05. (**C**) Heat map showing poly(A) tail length for all significant genes. Each cell corresponds to the sample-level standard deviation relative to the row mean. Red = high tail length, blue = low tail length. (**D**) Scatter plot comparing average tail length in control and differentiated cells, noting this sequenced tail length approximates the actual poly(A) tail length distributions. Diagonal line represents tail length parity. (**E**) Comparison of log_10_ gene length and change in poly(A) tail length. Pearson's correlation coefficient and associated *P*-values shown for genes with no significant change in tail length (grey) and increased tail length (red). (**F**) Bubble plot showing top 20 significantly enriched gene ontology terms for genes with significantly increased poly(A) tail length.

We next analysed changes in mRNA poly(A) tail length across 13021 genes with a detectable poly(A) tail. Across the transcriptome, average poly(A) tail length was significantly increased in differentiated cells (47.34 A residues) relative to controls (43.59 A residues), suggesting neuronal differentiation is associated with global poly(A) lengthening (*P* < 0.0001, Kolmogorov–Smirnov test; Figure 2A). Differential analysis of tail length identified 158 significantly altered genes, of which 156 (98.7%) exhibited poly(A) lengthening (Figure 2B & C, Table S3). On average, genes with significantly increased tail length gained ∼18 residues, compared to an average ∼3 residue increase for nonsignificant genes (Figure 2D). In view of recent evidence suggesting poly(A) tail length is negatively correlated with gene length in yeast (36,53,54), we analysed whether this was observable in our data, however no strong correlation was observed (Figure 2E). Finally, gene ontology (GO) analysis revealed genes with significantly increased tail length were predominantly associated with terms relating to neuronal differentiation, including *neurogenesis*, *generation of neurons*, *neuron differentiation* and *neuron development* (Figure 2F, Supplementary Table S4).

To explore the functional significance of polyadenylation during neuronal differentiation, we examined cellular properties after 5 days of ATRA treatment in the presence of cordycepin (CDY), an adenosine derivative known to prematurely terminate polyadenylation (55,56). A truncated differentiation paradigm was employed to capture changes during, rather than after, this phenotypic transition. Across three sub-IC_50_ (57) CDY concentrations (5μM, 10μM and 20μM), limited differences in gross cellular morphology were observed relative to cells treated with ATRA only (Supplementary Figure S3A). Furthermore, qPCR (See Supplementary Methods for further details) of six neuronal marker genes (*FOS*, *NGFR*, *SYN2*, *GRIK3*, *GRIA4* and *HOMER2*) revealed minimal changes after CDY treatment, except for *FOS* (log_2_FC = 0.486, *FDR* = 0.047) and *NGFR* (log_2_FC = 0.556, *FDR* = 0.047), which were upregulated in the 5μM CDY group relative to controls (Supplementary Figure S3B). Collectively, these results suggest that while poly(A) tail lengthening during neuronal differentiation predominantly affects genes associated with this phenotypic transition, pharmacological inhibition of polyadenylation appears to exert minimal impacts on neuronal differentiation.

### Increased poly(A) tail length is associated with enhanced mRNA expression

**Figure 3.**
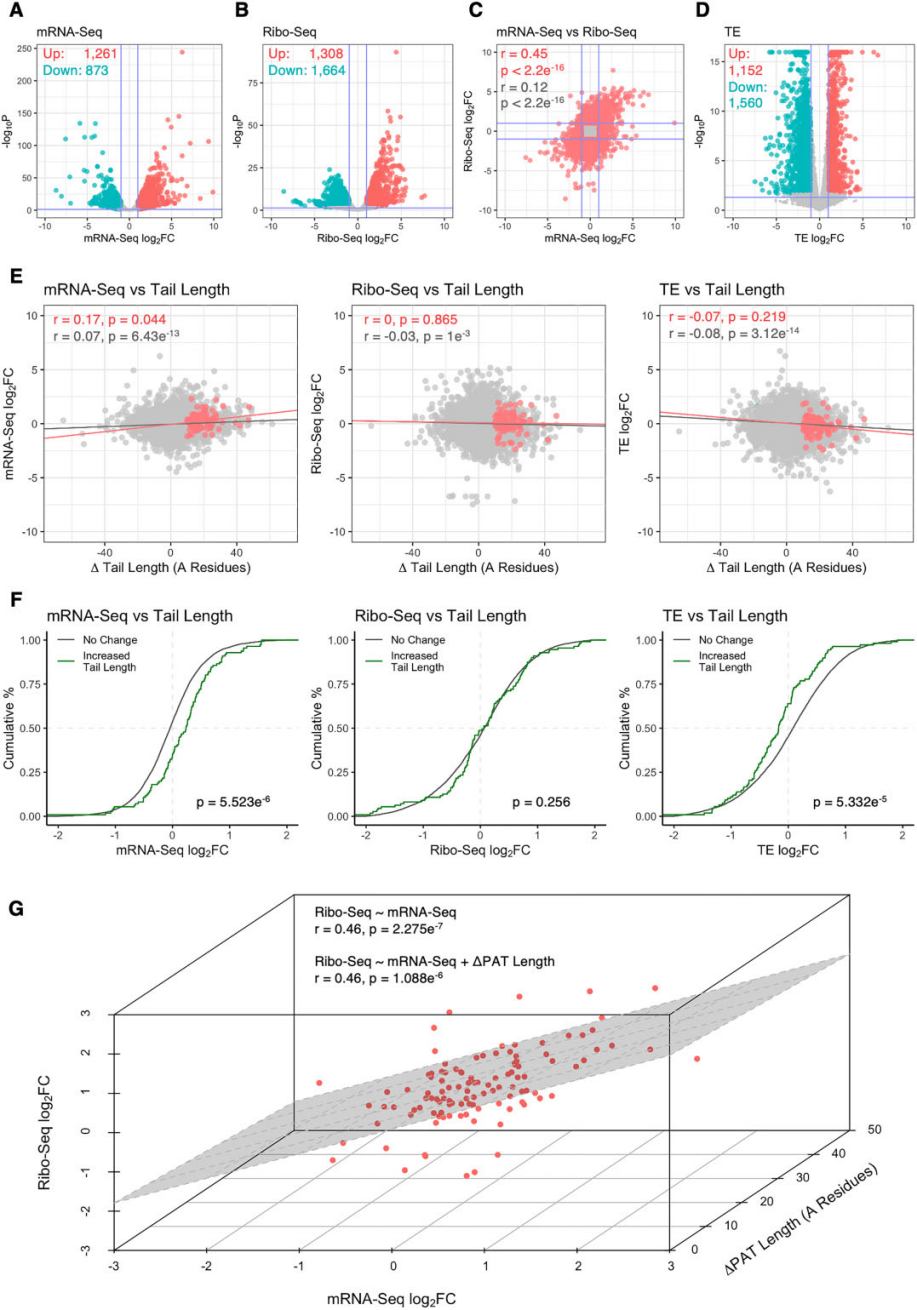
Interplay between poly(A) tail length, mRNA expression and translation. (**A**, **B**) Volcano plots comparing gene-level log_2_FC versus –log_10_*P* value for (A) mRNA-Seq and (B) Ribo-Seq libraries. Genes with significantly increased or decreased expression/translation are marked in red and blue, respectively, with Benjamini-Hochberg *FDR* < 0.05 and absolute log_2_FC > 1 considered significant. Horizontal blue line represents nominal *P* < 0.05. (**C**) Comparison of mRNA-Seq and Ribo-Seq log_2_FC values. Red = genes with significantly altered mRNA expression and/or translation, grey = all other genes. Pearson correlation coefficients and associated *P* values are reported top left. (**D**) As in (A, B), except depicting changes in translational efficiency. (**E**) Scatter plots comparing changes in tail length with mRNA expression, translation and translational efficiency. Genes with significantly increased tail length are marked in red. Pearson correlation coefficients and associated *P* values are reported top left. (**F**) Cumulative distribution of mRNA-Seq, Ribo-Seq and translational efficiency log_2_FC for genes with increased tail length (green) or no significant change (grey). Groups were compared via Kolmogorov–Smirnov test, with associated *P* values reported bottom right. (**G**) Three-dimensional scatter plot comparing changes in mRNA expression, translation and poly(A) tail length for genes with significantly increased tail length. Results for two linear models examining the effect of poly(A) tail length on the relationship between mRNA expression and translation are shown top left.

We next explored interplay between poly(A) tail length, mRNA expression and translation during neuronal differentiation. A total of 2134 differentially expressed and 2972 differentially translated genes were identified via mRNA-Seq and Ribo-Seq, respectively, with consistent enrichment of GO terms related to neuronal differentiation amongst upregulated genes in both datasets (Figure 3A, B, Supplementary Table S5-S8). The 2134 differentially expressed genes were validated by comparison with differential mRNA expression derived from the PAT-Seq data, which revealed strong correlation (*R*^2^ = 0.67, *P* < 2.2e^−16^) despite the expected differences in gene coverage (Supplementary Figure S4). Direct comparison of mRNA-Seq and Ribo-Seq log_2_FC values revealed positive correlation between changes in mRNA expression and translation for all differentially regulated genes (Pearson's *r* = 0.45, *P* < 2.2e^−16^_,_ Figure 3C). A further 2712 genes with significantly altered translational efficiency (TE) were also identified, which were enriched for a broad range of GO terms (Figure 3D, Supplementary Tables S9, S10).

For genes with significantly increased tail length, positive correlation was identified between poly(A) tail length and mRNA expression (*r*_mRNA_ = 0.17, *P* = 0.044), whereas translation and translational efficiency were not associated (*r*_RFP_ ≈ 0, *P* = 0.865, *r*_TE_ = –0.07, *P* = 0.219; Figure 3E). Similarly, a cumulative distribution analysis revealed genes with increased poly(A) tail length exhibited increased steady-state mRNA expression, versus genes without significant tail length shifts (Δlog_2_FC = 0.293, *P* = 5.523e^−6^, Kolmogorov–Smirnov test; Figure 3F). These same genes also exhibited a significant decrease in translational efficiency (Δlog_2_FC = –0.244, *P* = 5.332e^−5^; Figure 3F). In addition, linear models examining translational status as a function of mRNA expression amongst genes with increased tail length were not improved upon inclusion of poly(A) tail length as a covariate (*P* = 0.371, ANOVA; Figure 3G). Finally, GO analysis was conducted for 45 genes with significantly upregulated tail length and statistically elevated mRNA expression (*FDR*_*BH*_ < 0.05, log_2_FC > 0), revealing enrichment of key cellular component terms including *integral/intrinsic component of synaptic/postsynaptic membrane*, *synapse* and *dendritic spine* (Supplementary Table S11).

### Interplay between poly(A) tail dynamics and microRNA expression

**Figure 4.**
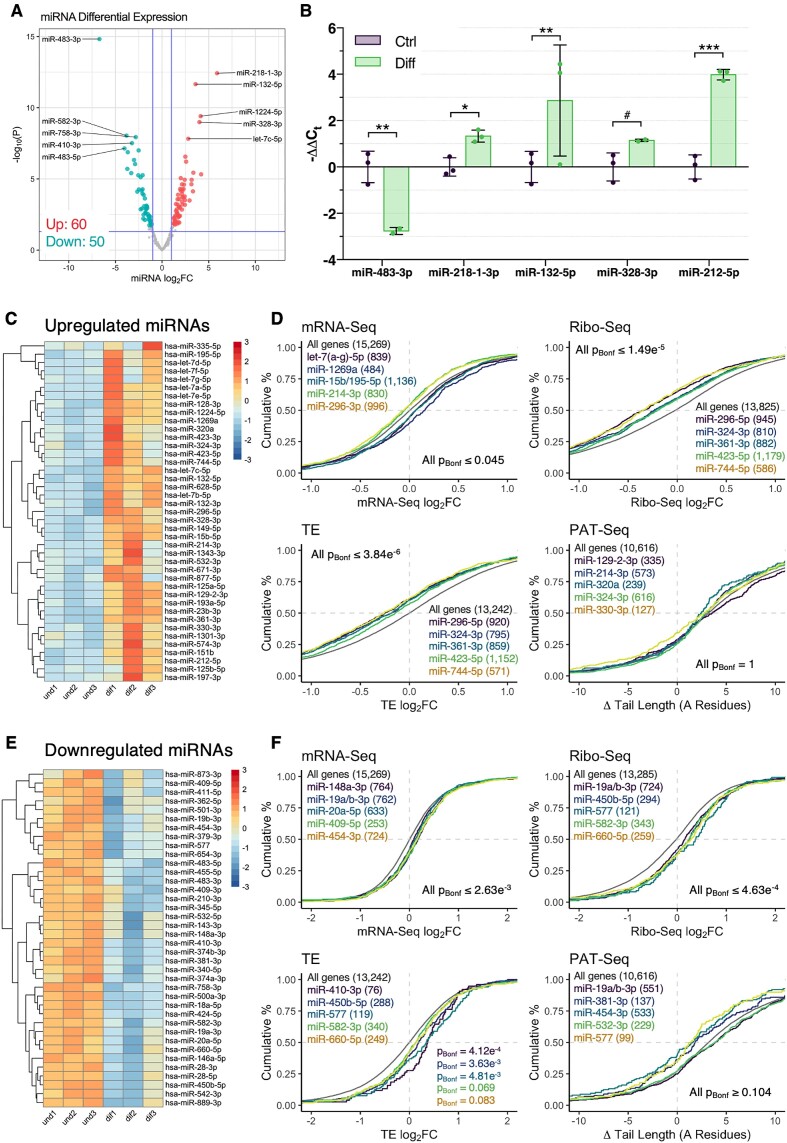
Differentiation-associated microRNAs are predominantly associated with changes in mRNA abundance and translation. (**A**) Volcano plot comparing log_2_FC and –log_10_*P* values of mature miRNAs differentially expressed during neuronal differentiation. MiRNAs with significantly increased or decreased expression are marked in red and blue, respectively, with Benjamini-Hochberg *FDR* < 0.05 and absolute miRNA log_2_FC > 1 considered significant. Horizontal blue line represents nominal *P* < 0.05. (**B**) Validation of five miRNAs via qPCR. ΔCt values between groups were compared via Student's *t*-test, with post-hoc correction for multiple testing via Benjamini, Krieger and Yekutieli method (^#^*FDR* < 0.1, *FDR < 0.05, **FDR < 0.01, ***FDR < 0.001). Data presented as mean -ΔΔC_t_ ± SD. (**C**) Heat map depicting expression of 41 significantly upregulated and confidently annotated miRNAs. Each cell corresponds to the sample-level log_2_CPM standard deviation relative to the row mean. Red = high expression, blue = low expression. (**D**) Cumulative distribution of mRNA expression, translation, translational efficiency and poly(A) tail length for genes targeted by upregulated miRNAs. For each miRNA, target genes were compared to non-targets via Kolmogorov–Smirnov test, with Bonferroni-corrected *P* values and number of genes per group reported. Results for the top 5 miRNAs (ranked by *P*_*Bonf*_) are depicted. Grey line depicts the transcriptome for reference. (**E**) As in (C), except depicting the 38 downregulated and confidently annotated miRNAs. (**F**) As in (D), except analysing targets of downregulated miRNAs.

miRNA expression during neuronal differentiation was next examined to explore miRNA-associated poly(A) tail modulation (14–16). In total, 110 differentially expressed mature miRNAs were identified via small RNA sequencing, including upregulation of miRNAs previously associated with neuronal differentiation such as miR-132–5p and miR-212–5p (58) (Figure 4A; Supplementary Table S12). Five miRNAs selected for validation via qPCR (miR-483–3p, miR-218–1-3p, miR-132–5p, miR-328–3p and miR-212–5p) exhibited directionally consistent differential expression, noting that all miRNAs were statistically significant (*FDR* < 0.05), except for miR-328–3p, which trended towards differential expression (*FDR* = 0.079, Figure 4B).

The behaviour of mRNAs targeted by differentially expressed miRNAs was explored using *in silico* miRNA–mRNA predictions from TargetScan v7.2 (59), excluding 31 miRNAs deemed potentially misannotated by the TargetScan database, as well as low confidence mRNA–miRNA pairings with cumulative weighted context++ score > –0.2. Genes targeted by upregulated miRNAs exhibited decreased translation and translational efficiency, however no consistent changes in mRNA expression nor poly(A) tail length survived correction for multiple testing (Figure 4C, D, top 5 most significant miRNA target gene profiles shown). Similarly, genes targeted by downregulated miRNAs displayed increased mRNA expression, translation and translational efficiency, but no significant alteration of poly(A) tail length (Figure 4E, F). These findings were largely consistent after analysing the combined effect of all upregulated or downregulated miRNAs, stratified the number of predicted miRNA recognition elements for each gene (Supplementary Figure S5). We also investigated post-transcriptional dynamics associated with tRNA-derived small RNA fragments, a class of short non-coding RNA related to miRNAs (60,61), however no consistent changes were observed (Supplementary Figure S6).

**Figure 5.**
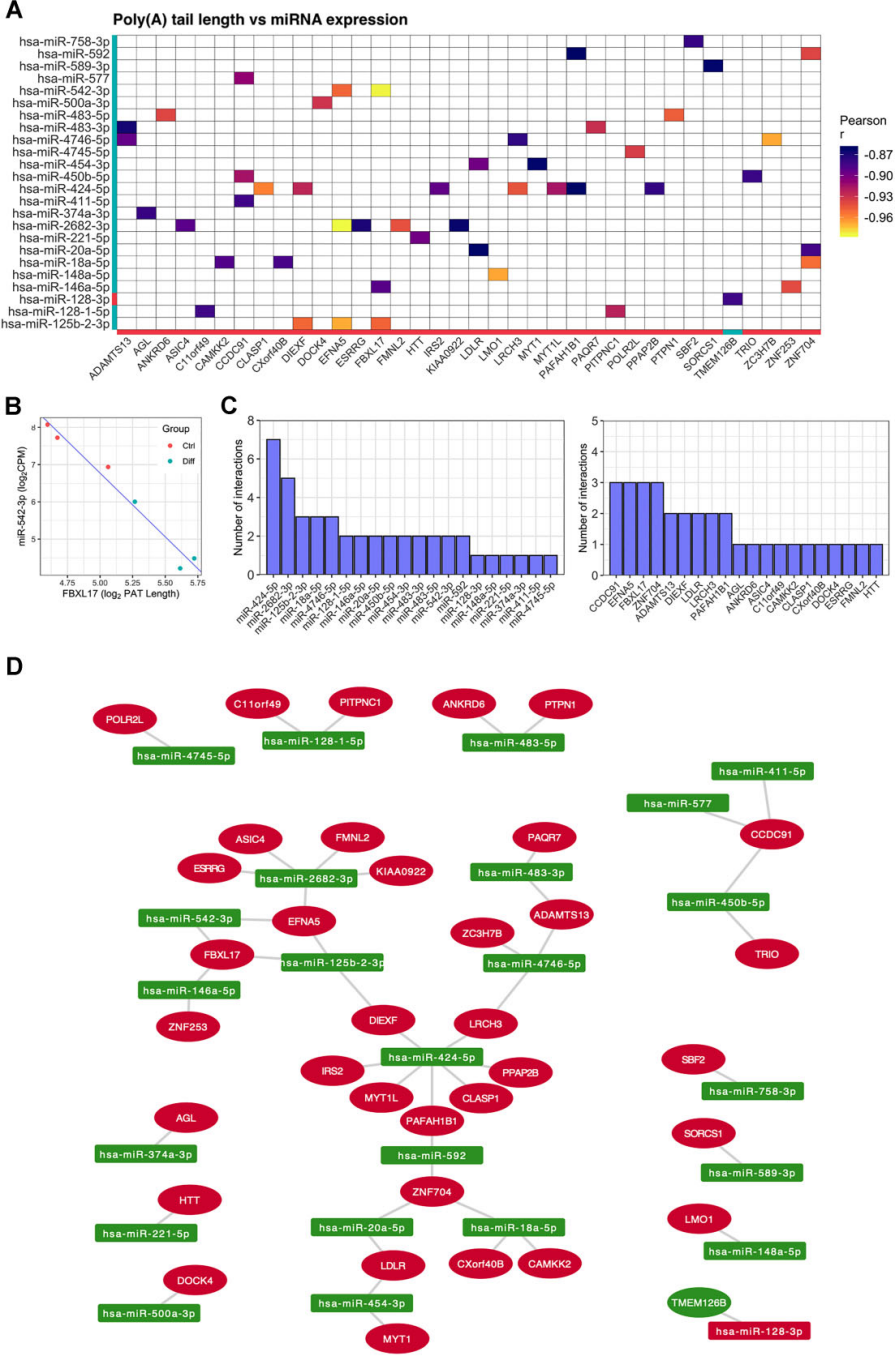
Identification of miRNA–mRNA correlations associated with changes in mRNA poly(A) tail length. (**A**) Heat map showing all 49 significant negative correlations between genes with significant changes in poly(A) tail length and miRNAs. Red bars = increased expression/tail length, blue bars = decreased expression/tail length. (**B**) Scatter plot showing the most significant negative mRNA–miRNA correlation. (**C**) The top 20 most frequently represented miRNAs (left) and mRNAs (right) in the correlation analysis. **(D)** Network diagram showing all negative correlations presented in panel (A). Green nodes = increased expression/tail length, red nodes = decreased expression/tail length.

To probe specific miRNA-poly(A) tail length associations, negatively correlated miRNA–mRNA pairings were analysed with *miRComb* (62). We identified 49 significant correlations between 24 differentially expressed miRNAs and 36 mRNAs (*FDR*_*BH*_ < 0.05; Figure 5A, see Figure 5B for top miRNA–mRNA pairing, see Figure 5C for number of correlations for each miRNA and mRNA). These genes exhibited enrichment for a diverse range of GO terms including *sorting endosome*, *establishment of mitotic spindle localization* and *positive regulation of embryonic development* (Supplementary Tables S13, S14). Interestingly, a subnetwork consisting of 22 genes and 11 miRNAs was uncovered, however these genes exhibited no significant enrichment of GO annotations (Figure 5D). Collectively, these findings suggest that while miRNA expression is generally associated with mRNA expression and translation during differentiation, any observable effect on poly(A) tail length may be confined to specific miRNA–mRNA pairings in our paradigm.

### Increased usage of distal polyadenylation signals during neuronal differentiation

**Figure 6.**
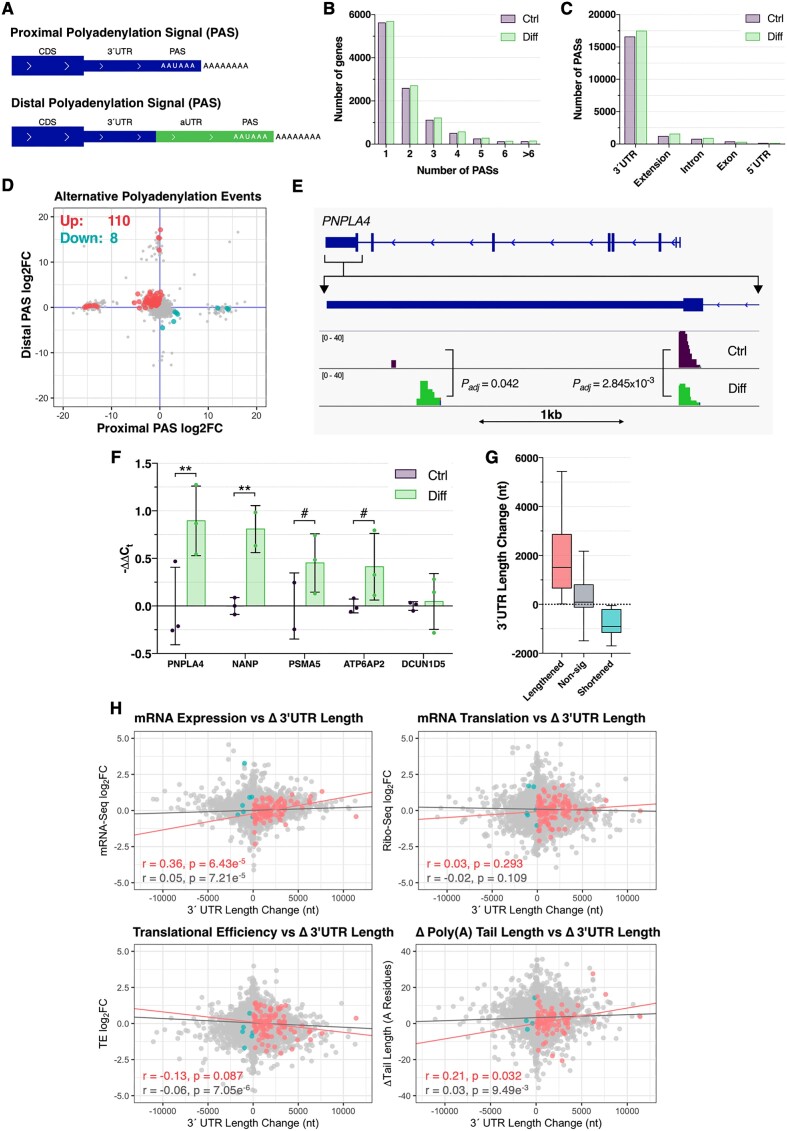
Differentiation-associated alternative polyadenylation. (**A**) Schematic representation of alternative polyadenylation (APA). (**B**) Number of confidently detected (≥2 normalised counts in three biological replicates) polyadenylation signals (PAS) per gene, noting that PAS distributions did not significantly differ between conditions (*P* = 0.195, Chi-squared test). (**C**) Distribution of PAS amongst genomic regions. Note that ‘extensions’ refer to PAS located downstream from the most 3′ exon, while ‘exonic’ PAS refer to coding exons. PAS distributions were found to significantly differ between conditions (*P*< 0.0001, Chi-squared test). (**D**) Scatter plot comparing the log_2_FC of the top 2 most expressed APA sites per gene. Genes with a statistically significant (Benjamini-Hochberg *FDR* < 0.05) change for at least one PAS and an absolute relative expression difference (RED) between proximal and distal sites >1 were considered significant. Red points = genes with significant lengthening events, blue points = genes with significant shortening events. (**E**) PAT-Seq read depth for the *PNPLA4* gene, depicting a significant increase for the distal PAS, and decrease for the proximal PAS after neuronal differentiation. (**F**) qPCR analysis of 5 genes with significant APA events. Two primer sets were used to examine expression of the alternative 3′UTR versus a common upstream region. ΔCt values between groups were compared via Student's *t*-test, with post-hoc correction for multiple testing via Benjamini, Krieger and Yekutieli method (^#^*FDR* < 0.1, **FDR < 0.01). Data presented as mean –ΔΔC_t_ ± SD. (**G**) Box plots comparing 3′UTR length changes for genes with significant lengthening events (red), shortening events (blue) or no significant change (grey). (**H**) Scatter plots comparing 3′UTR length changes with mRNA expression, translation, translational efficiency and poly(A) tail length changes. Genes with significantly increased or decreased 3′UTR length are marked in red and blue, respectively. Pearson correlation coefficients and associated *P* values reported bottom left.

Recent studies suggest alternative polyadenylation is highly dynamic during long-term cellular processes such as differentiation (32–35) (Figure 6A). To establish whether alternative polyadenylation (APA) was prevalent during neuronal differentiation, changes in polyadenylation signal (PAS) usage were determined from the PAT-Seq data using *DEXSeq* (41). Overall, 22898 PASs across 11213 genes were identified, with more than 45% of genes containing > 1 expressed PAS (Figure 6B). Approximately 87% PASs were identified within 3′UTRs as anticipated, whereas ∼7% were downstream of the most 3′ exon (‘extensions’), and a further ∼4% expressed from intronic regions (Figure 6C). Notably, the genomic distribution of expressed PASs significantly differed between both conditions (*P* < 0.0001, Chi-squared test), indicating potential for differential regulation during neuronal differentiation (Figure 6C).

Gene level changes in PAS usage were next examined to identify significant APA events. To simplify this analysis, the top two most expressed PAS were analysed for each gene, noting that we predominantly focused on 3′UTR PASs, which possess the highest capacity to modulate miRNA binding sites and other regulatory elements. Differential analysis revealed 118 genes with significant APA events, of which 110 (93%) were associated with 3′UTR lengthening (Figure 6D, see Figure 6E for representative lengthening event, Supplementary Table S15). Five genes were selected for qPCR validation using primer sets targeting the alternative 3′UTR and a common upstream region, of which two (*PNPLA4*, *NANP*) were significantly lengthened (*FDR* < 0.01) and two (*PSMA5*, *ATP6AP2*) trended towards lengthening (*FDR* = 0.074, Figure 6F). Quantification of 3′UTR length changes revealed lengthened genes were subjected to a median increase of 1,517nt, whereas shortened genes exhibited a median decrease of 903nt (Figure 6G). In contrast, the remainder of the transcriptome increased by a median of 100nt (Figure 6G). Surprisingly, GO term analysis revealed no consistent patterns of enrichment upon analysis of significantly lengthened and shortened genes, indicating genes affected by APA events were functionally diverse (Supplementary Table S16).

### Effect of 3′UTR lengthening on mRNA dynamics and miRNA binding sites

Since 3′UTR length changes are thought to impact critical regulatory motifs (24–26), the effect of APA events on mRNA expression, translation, and poly(A) tail length were explored. These analyses were restricted to significant lengthening events, as shortening events impacted an insufficient number of genes for reliable statistical analysis. Overall, 3′UTR lengthening was significantly correlated with mRNA expression (*r* = 0.36, *P* = 6.43 × 10^−5^) and poly(A) tail length (*r* = 0.21, *P* = 0.032), but not mRNA translation (*r* = 0.03, *P* = 0.293) or translational efficiency (*r* = –0.13, *P* = 0.087; Figure 6H). Interestingly, genes with lengthened 3′UTRs were also subjected to increased poly(A) tail length, however the magnitude of poly(A) lengthening was lower than genes unaffected by APA (*P* = 0.037; Supplementary Figure S7). Furthermore, the top 50% of lengthened genes were biased towards increased mRNA expression, whereas the lower 50% of genes exhibited decreased expression (*P*_Bonferroni_ ≤ 0.0219 for all comparisons, Kruskal–Wallis test with Dunn's post-hoc multiple comparisons test; Supplementary Figure S7). Together, these results suggest that 3′UTR extensions via APA during neuronal differentiation are associated with mRNA expression and poly(A) tail length.

**Figure 7.**
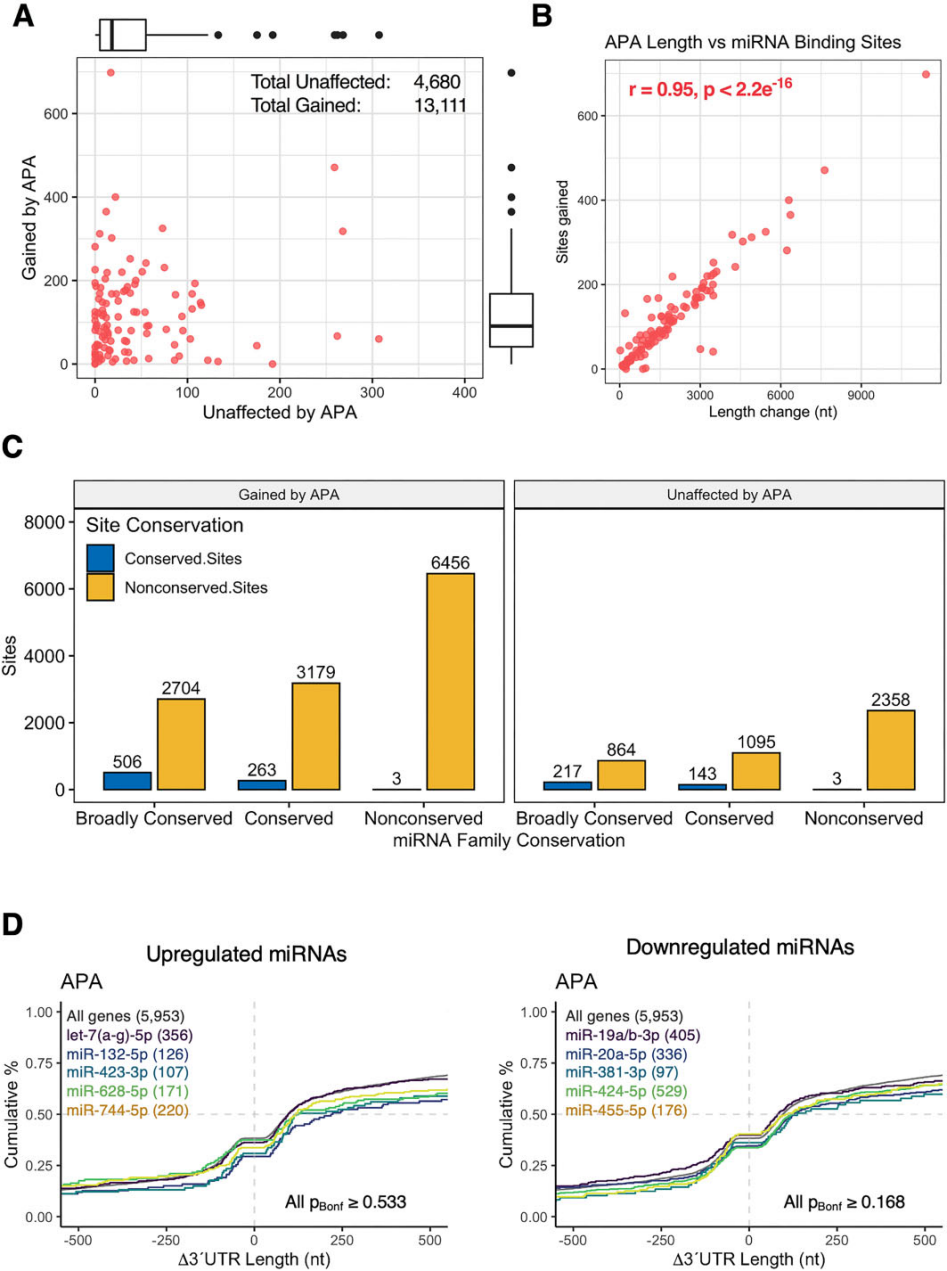
The effect of 3′UTR lengthening on miRNA binding sites. (**A**) Scatter plot comparing the number of miRNA binding sites unaffected by or gained by differentiation-associated lengthening events. (**B**) Comparison of 3′UTR length changes and the number of miRNA binding sites gained by significantly lengthened genes. Pearson's correlation coefficient and associated *P* value are reported top left. (**C**) The number of miRNA binding sites affected and unaffected by 3′UTR lengthening events, stratified by binding site and miRNA evolutionary conservation. A significant difference between conserved and non-conserved sites was identified for sites gained by APA (*P* < 2.2e^−16^, Chi-squared test). Similarly, conservation profiles significantly differed between sites gained by or unaffected by APA (*P* = 2.989e^−6^). (**D**) Cumulative distribution of 3′UTR length changes for genes targeted by upregulated and downregulated miRNAs. Groups were compared to non-target genes via Kolmogorov–Smirnov test, with the top 5 miRNAs ranked by Bonferroni-corrected *P* value depicted.

We next examined whether 3′UTR lengthening events impacted the expression of miRNA binding sites. In total, 3′UTR lengthening resulted in the expression of 13111 new miRNA binding sites across 110 genes (median 92 per gene), whereas 4680 binding sites were unaffected by APA events (median 18 per gene), indicating 3′UTR lengthening considerably altered expression of miRNA recognition elements (Figure 7A, Supplementary Table S17). The number of miRNA binding sites gained by APA was strongly correlated with 3′UTR length changes as anticipated (*r* = 0.95, *P* < 2.2e^−16^; Figure 7B). Strikingly, non-conserved binding sites (12339, 94%) and non-conserved miRNAs (6456, 49%) were most frequently affected by 3′UTR lengthening (*P* < 2.2e^−16^, Chi-squared test), and these frequencies significantly differed upon comparison with sites unaffected by APA (*P* = 2.989e^−6^; Figure 7C). Interestingly, 986 (7%) and 1085 (8%) gained sites were associated with significantly upregulated and downregulated miRNAs, respectively, with miR-532–3p the most affected upregulated miRNA (55 sites) and miR-340–5p the most affected downregulated miRNA (71 sites; Supplementary Table S18). However, genes targeted by upregulated and downregulated miRNAs exhibited no significant bias towards lengthening or shortening compared to the transcriptome (Figure 7D).

### Differential expression and translation of genes associated with polyadenylation

**Figure 8.**
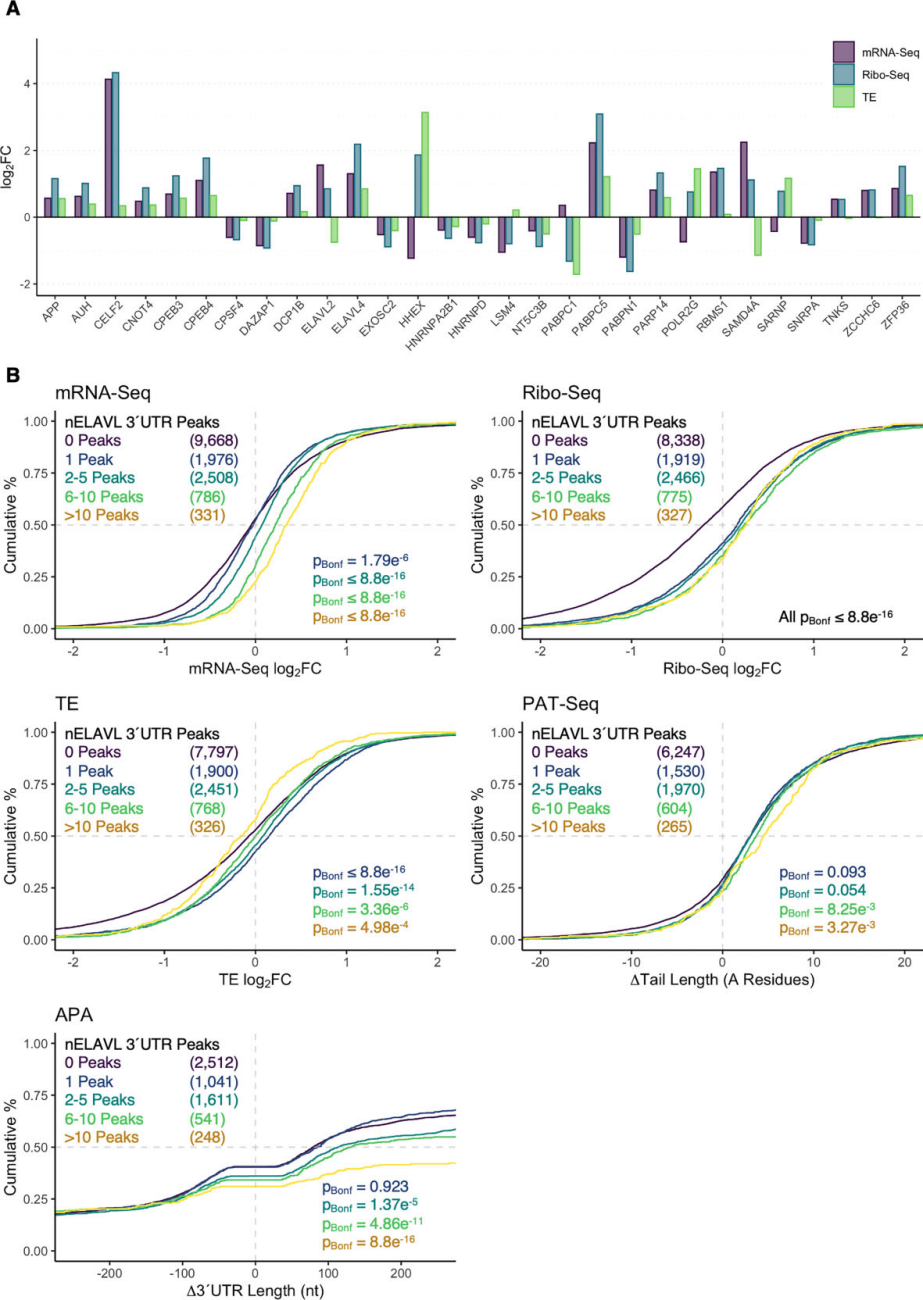
Differential expression of genes associated with regulation of polyadenylation. (**A**) Changes in mRNA expression, translation and translational efficiency for 29 effectors annotated to gene ontology terms associated with polyadenylation and poly(A) tail regulation (MSigDB). All depicted changes in mRNA-Seq and Ribo-Seq log_2_FC were statistically significant (*FDR* < 0.05). (**B**) Cumulative distribution of mRNA expression, translation, translational efficiency, poly(A) tail length and 3′UTR length changes for genes with 3′UTR CLIP-Seq peaks specific to neuronal ELAV-like (nELAVL) RNA binding proteins. All groups were compared to the transcriptome via Kolmogorov–Smirnov test, with associated Bonferroni-corrected *P* values reported bottom right. The number of genes per group is reported top left. CLIP-Seq peaks were obtained from Scheckel *et al.* (65).

We next examined the expression and translation of genes potentially associated with regulation of poly(A) tail and 3′UTR length during neuronal differentiation. Utilising GO annotations associated with cleavage and polyadenylation and deadenylation (MSigDB (63)), 29 genes were identified with significant changes at the mRNA and translational levels (Figure 8A). Notably, none of these 29 genes exhibited significant alteration of poly(A) tail length, nor APA. Interestingly, robust transcriptional and translational upregulation was identified for *CELF2*, a known regulator of alternative polyadenylation events (64). Dual upregulation was also observed for the Poly(A)-Binding Protein C5 (*PABPC5*), brain-enriched Cytoplasmic Polyadenylation Element Binding Proteins *CPEB3* and *CPEB4*, and neuron-specific ELAV-like RNA Binding Proteins *ELAVL2* and *ELAVL4*. Since neuronal ELAVL (nELAVL) proteins have been previously associated with multiple aspects of mRNA metabolism (65), we examined the behaviour of nELAVL target genes utilising CLIP-Seq data from human brain samples (65). Interestingly, the number of nELAVL 3′UTR peaks exhibited a cumulative relationship with mRNA expression (*P*_Bonferroni_ ≤ 1.79e^−6^, Kolmogorov–Smirnov test), while genes with ≥1 nELAVL site were subjected to translational enhancement versus genes with no site (*P*_Bonferroni_ ≤ 8.8e^−16^; Figure 8B). Furthermore, higher levels of nELAVL peaks were associated with poly(A) tail (*P*_Bonferroni_ ≤ 8.25e^−3^ for genes with ≥ 6 peaks) and 3′UTR lengthening (*P*_Bonferroni_ ≤ 1.37e^−5^ for genes with ≥2 peaks, Figure 8B).

## DISCUSSION

The complex and dynamic network architecture in the brain is established and regulated though its neuronal nodes. These features are made possible by the discrete temporospatial control of RNA translation that is mediated by a plethora of interacting post-transcriptional regulatory mechanisms. In the current study, we explored the interplay between several of these processes during neuronal differentiation using a variety of RNA sequencing strategies, to characterize mRNA 3′UTR dynamics and their interrelationship with mRNA abundance, translation, and miRNA. Our findings suggest modulation of poly(A) tail length and APA function as part of a rich regulatory matrix, enabling dynamic coordination of mRNA during differentiation.

Transcriptome-wide quantification of poly(A) tail length revealed a bias towards poly(A) lengthening during neuronal differentiation, which predominantly affected genes enriched for differentiation-associated functions. Interestingly, many genes with increased poly(A) tail length also exhibited upregulation of steady-state mRNA levels in a manner which disproportionately affected genes annotated to the synapse, whereas translational profiles for the same genes remained largely unchanged. The expression of long poly(A) tails in eukaryotic cells is known to support association with poly(A) binding proteins, which establish 5′ to 3′ mRNA interactions important for mRNA stability and translational activity (12,13,66). While changes in polyadenylation and their relationship with mRNA expression have not been observed previously during neuronal differentiation, recent studies have shown that poly(A) tail lengthening in developing oocytes enhances stability and translation of mRNAs associated with embryonic development (53,67,68). Strikingly, emerging evidence suggests poly(A) tail length and cytoplasmic poly(A) binding protein, *PABPC*, function as key mediators of translational enhancement in oocytes and early embryos, whereas mRNA stability is favoured in comparatively mature cell types, underscoring the importance of polyadenylation as a critical developmental switch (69). These studies are consistent with the observed association between poly(A) tail lengthening and mRNA expression, but not translation, in the current study. It is plausible that poly(A) tail lengthening may exert a more-robust effect on neuronal translation in response to acute stimuli such as neuronal activation, as observed previously in paradigms of long-term potentiation (17,70). Nonetheless, we suspect poly(A) tail lengthening may contribute to neuronal differentiation by enhancing the abundance and stability of mRNAs important for this phenotypic transition, however further exploration is required to dissect the significance of poly(A) lengthening and increased mRNA expression in the absence of enhanced translational activity.

Analysis of miRNA expression revealed substantial alteration of miRNA profiles during neuronal differentiation, including upregulation of neuronally enriched miRNAs, such as miR-132–5p and miR-212–5p, consistent with previous analysis of ATRA/BDNF differentiated SH-SY5Y cells (58). Interestingly, genes targeted by differentially expressed miRNAs were subjected to altered mRNA expression, translation and translational efficiency in a manner directionally consistent with the canonical model of miRNA function, characterised by sequential deadenylation, decapping and exonucleolytic degradation of target mRNAs (14–16). Similar profiles of miRNA-associated mRNA repression have been previously observed on a transcriptome-wide scale after repeated depolarisation of neuronally differentiated cells (71). Contrary to expectation, however, associations between miRNA and poly(A) tail length were restricted to a small subset of miRNA–mRNA correlations. We suspect transcriptome-wide miRNA-dependent regulation of the poly(A) tail may be more readily observed during early stages of neuronal differentiation, during which post-transcriptional remodelling of mRNA temporospatial dynamics is likely to be prevalent, thus necessitating future studies with greater temporal resolution.

A clear bias towards 3′UTR lengthening was additionally uncovered, consistent with previous studies of neuronal differentiation and development (32–35). Interestingly, the diverse functional representation of lengthened genes has also been observed in a subset of these studies (32,33), suggesting that discrete APA events have functional significance at the individual gene level. We also identified positive correlation between 3′UTR lengthening, mRNA expression and poly(A) tail length, a surprising finding given that 3′UTR lengthening is thought to enhance the inclusion of *cis*-acting regulatory elements associated with mRNA destabilization, particularly AU-rich elements and miRNA binding sites (23,24,26). Indeed, over 13000 miRNA binding sites were gained via 3′UTR lengthening in the current study. While this contradicts the canonical mechanism of miRNA function, it is plausible that many of these sites do not conform to conventional mechanisms of miRNA repression, or are not well adapted, given the vast majority of affected sites were non-conserved and affected non-conserved miRNA families (71). This consequently represents an exciting avenue for further mechanistic exploration, in which key miRNAs uncovered in the current study – such as miR-532–3p and miR-340–5p – may yield novel insights. We also cannot exclude the possibility that APA-associated changes in 3′UTR length may serve to control the subcellular localisation of affected mRNAs in differentiated neurons, considering localisation elements are predominantly confined to the 3′UTR (72). Finally, we note that APA sites identified from the mRNA-Seq data using *DaPars2* (28) exhibited weak correlation with APA events identified from the PAT-Seq data (Pearson *r* = 0.056, *P* = 1.1 × 10^–4^, Supplementary Figure S8, Table S19). Furthermore, only one of five *DaPars2*-nominated sites selected for qPCR was successfully validated, suggesting 3′-enriched sequencing methods may exhibit greater specificity for APA analysis than conventional mRNA-Seq (Supplementary Figure S8, Table S19).

Interestingly, several genes involved in regulation of pre-mRNA cleavage, alternative polyadenylation and poly(A) tail binding were differentially expressed and translated in the current study, which may underpin the observed changes in 3′UTR regulation. Notably, we identified transcriptional and translation upregulation of the neuron-specific *ELAVL2* and *ELAVL4* genes, members of the ELAVL family of AU-rich element RNA binding proteins associated with neuronal differentiation and development (73,74), as well as mRNA stability, translation, polyadenylation and splicing (65,75). Strikingly, experimentally supported nELAVL target genes exhibited significantly altered mRNA expression, translation, poly(A) tail length and 3′UTR length in the current study, consistent with previous evidence (65,75). We additionally identified robust upregulation of *CELF2* and *PABPC5*, both of are associated with expression of distal PAS and may therefore contribute to the 3′UTR lengthening observed in this study (64,76,77). However, some differentially expressed genes associated with polyadenylation did not change in a manner consistent with recent findings. A major example is *PABPN1*, a gene strongly associated with polyadenylation efficiency by tethering cleavage and polyadenylation specificity factor (CPSF) with poly(A) polymerase during poly(A) tail extension (78). This effector also promotes usage of distal PAS by binding proximal PAS and preventing their cleavage (76,78,79). Despite these findings, significant downregulation of CPSF was observed during differentiation, suggesting if this gene contributes to the patterns of 3′UTR and poly(A) tail extension observed during neuronal differentiation, its functional status circumvents its mRNA levels. Likewise, no significant changes were observed for any poly(A) polymerase genes, suggesting poly(A) tail length modulation was not a consequence of altered poly(A) polymerase expression.

In summary, our study suggests that mRNA 3′UTR and poly(A) tail length regulation is prevalent during neuronal differentiation and exhibits varying degrees of association with mRNA expression, translation and miRNA. Since 3′ mRNA features encode significant regulatory capacity, we suspect alternative polyadenylation and poly(A) tail length modulation contribute substantially to the unique spatial and temporal distribution of gene expression in neurons. However, further mechanistic analysis of these systems will be required to fully elucidate their functional significance in the neuronal context under normal and pathological conditions.

## Supplementary Material

gkad499_Supplemental_Files

## Data Availability

All raw and processed sequencing data are currently available online at the Gene Expression Omnibus (GEO), accession number GSE155432.

## Notes

Present address: Murray J. Cairns, Medical Sciences, The University of Newcastle, Callaghan, NSW 2308, Australia.
