# Supplementary material for: Extension of mRNA poly(A) tails and 3′UTRs during neuronal differentiation exhibits variable association with post-transcriptional dynamics
Source: Nucleic Acids Res. 2023 Jun 9;51(15):8181–98. doi: 10.1093/nar/gkad499 (PMC10450200; doi:10.1093/nar/gkad499)
Supplement: gkad499_Supplemental_Files [file gkad499_supplemental_files.zip › supplementary_methods_and_figures_dkiltschewskij_NAR_19052023_resubmission.pdf]

Extension of mRNA Poly(A) Tails and 3´UTRs During Neuronal Differentiation Exhibits Variable Association with Post-transcriptional Dynamics

Supplementary Material

SUPPLEMENTARY FIGURES

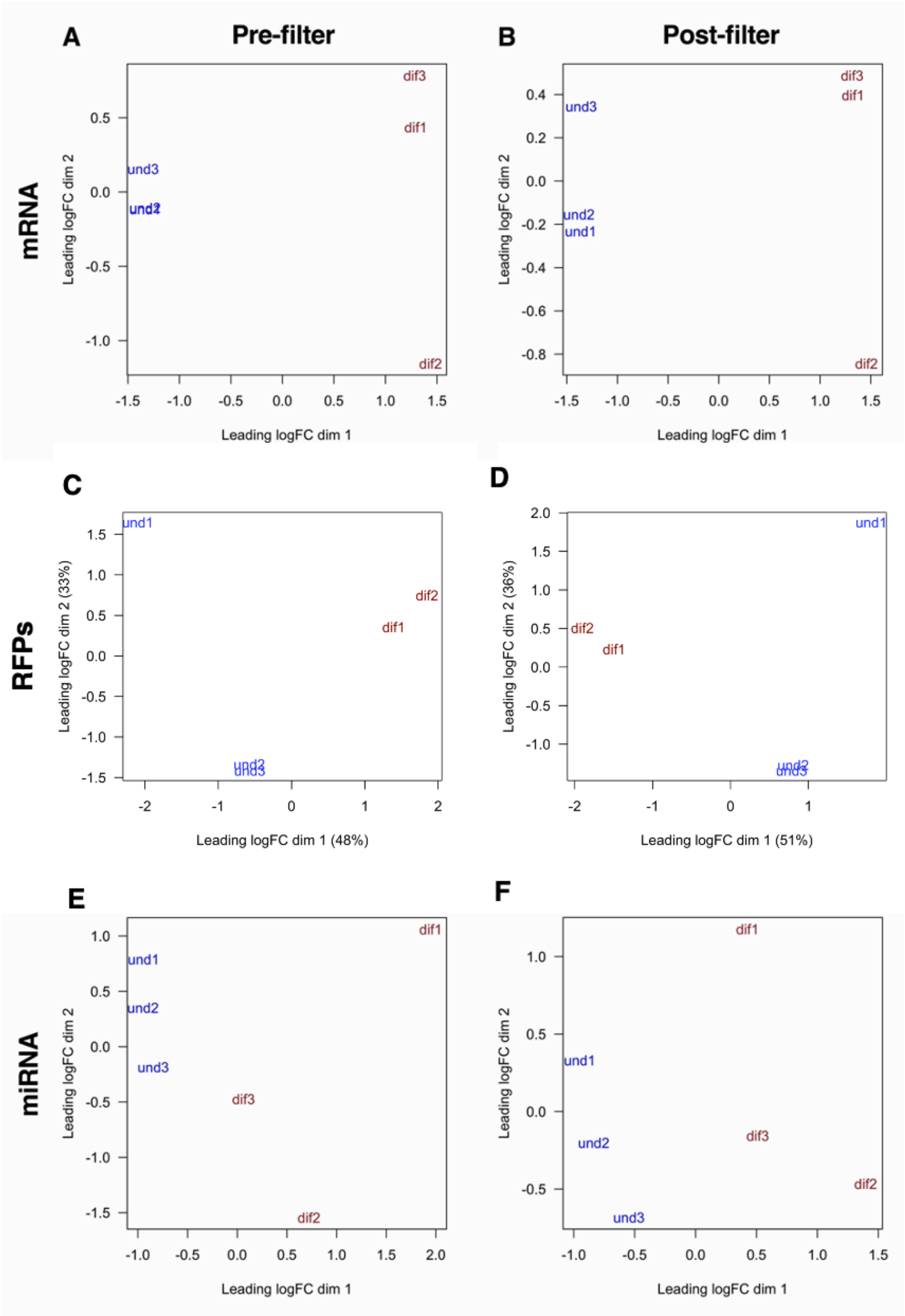

Supplementary Figure 1. Multidimensional Scaling of normalised read-counts. Multidimensional scaling (MDS) plots for mRNA-Seq **(A,B)**, Ribo-Seq **(C,D)** and small RNA-Seq **(E,F)** data before **(A,C,E)** and after **(B,D,F)** filtration of lowly expressed mRNAs and miRNAs.

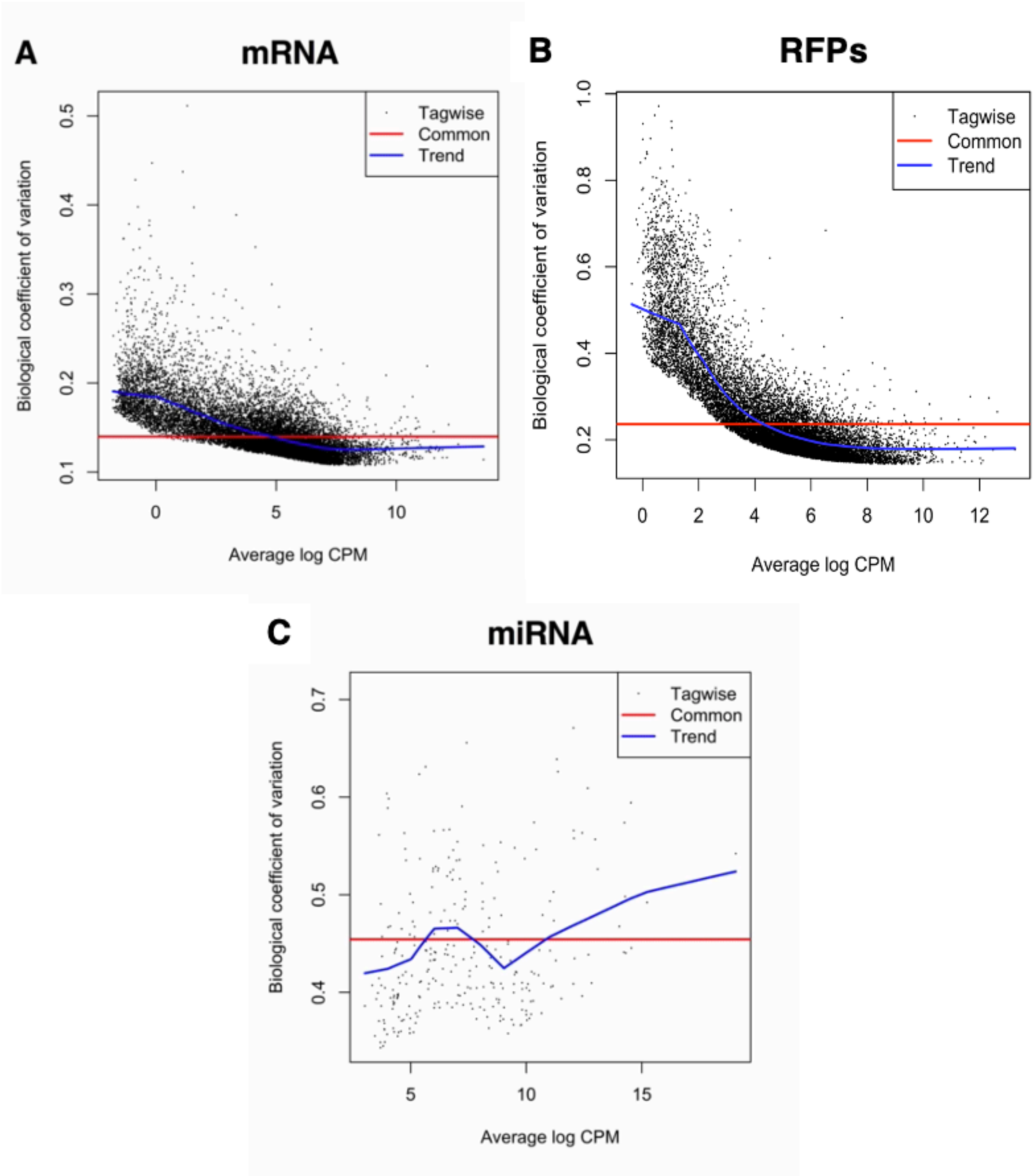

Supplementary Figure 2. Assessment of biological variation. Plots depicting biological coefficients of variation (BCV) vs average $\log_2$ counts per million (CPM) for all genes **(A,B)** and miRNAs **(C)**. For genes, biological variation was found to decrease as expression increased, as indicated by the decreasing blue trend line. In contrast, more variable biological variation was detected amongst miRNAs.

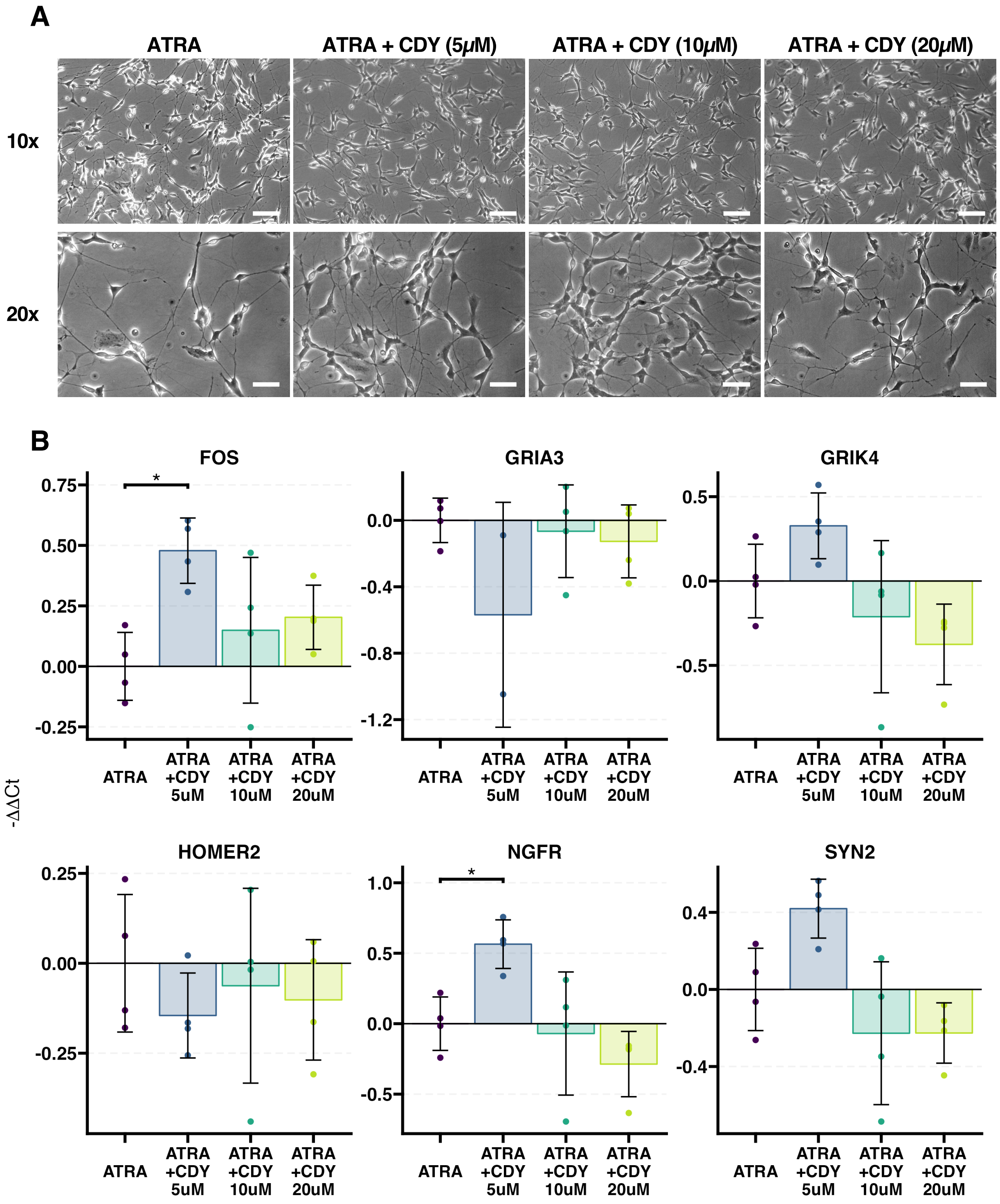

Supplementary Figure 3. Effect of cordycepin on SH-SY5Y morphology and expression of neuronal marker genes during neuronal differentiation. (A) Phase contrast micrographs depicting SH-SY5Y cells 5 days after media supplementation with *all-trans* retinoic acid (ATRA, 10µM) only, or ATRA plus cordycepin (CDY, also known as 3-deoxyadenosine), an adenosine analogue known to promote premature termination of polyadenylation. Cells were treated with either 0µM (controls), 5µM, 10µM or 20µM CDY as indicated, with culture medium and supplements (i.e. ATRA and CDY) replaced daily. Objective magnification indicated left. Scale bars represent 100µm (top) and 50µm (bottom). Note that a truncated differentiation paradigm was employed (i.e. *sans* BDNF and serum starvation) to capture changes during the most intensive period of

phenotypic change. **(B)** Bar plots comparing the expression of six neuronal marker genes (*FOS*, *GRIA3*, *GRIK4*, *HOMER2*, *NGFR* and *SYN2*) between CDY-treated cells and ATRA-only controls, as determined via RT-qPCR. Note that only *FOS* (log_2FC = 0.486, FDR = 0.047) and *NGFR* (log_2FC = 0.556, FDR = 0.047) exhibited a statistically significant change upon CDY cotreatment, both of which were observed for the 5μM group. ΔCt values between control and CDY-treated cells were compared via Student's *t*-test, with Benjamini, Kreiger and Yekutieli two-stage linear step-up procedure utilised to correct for multiple testing across all comparisons (* = FDR < 0.05). All samples were normalised to the geometric mean of *GUSB* (β-glucuronidase) and *TUBB2A* (tubulin β-2A) reference genes. Data presented as mean –ΔΔCt ± SD.

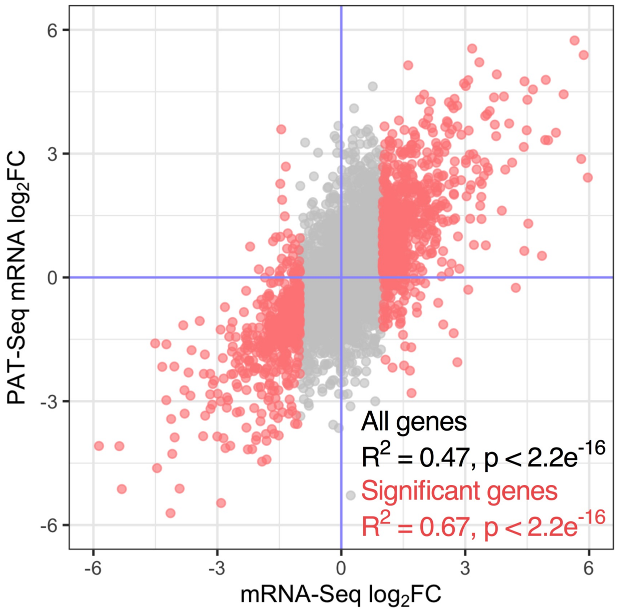

Supplementary Figure 4. Validation of mRNA-Seq expression. Scatter plot depicting mRNA log_2FC values computed via mRNA-Seq (x axis) and PAT-Seq (y axis) library preparation methods. Significantly differentially expressed genes detected by mRNA-Seq (red) were strongly correlated with PAT-Seq level expression data.

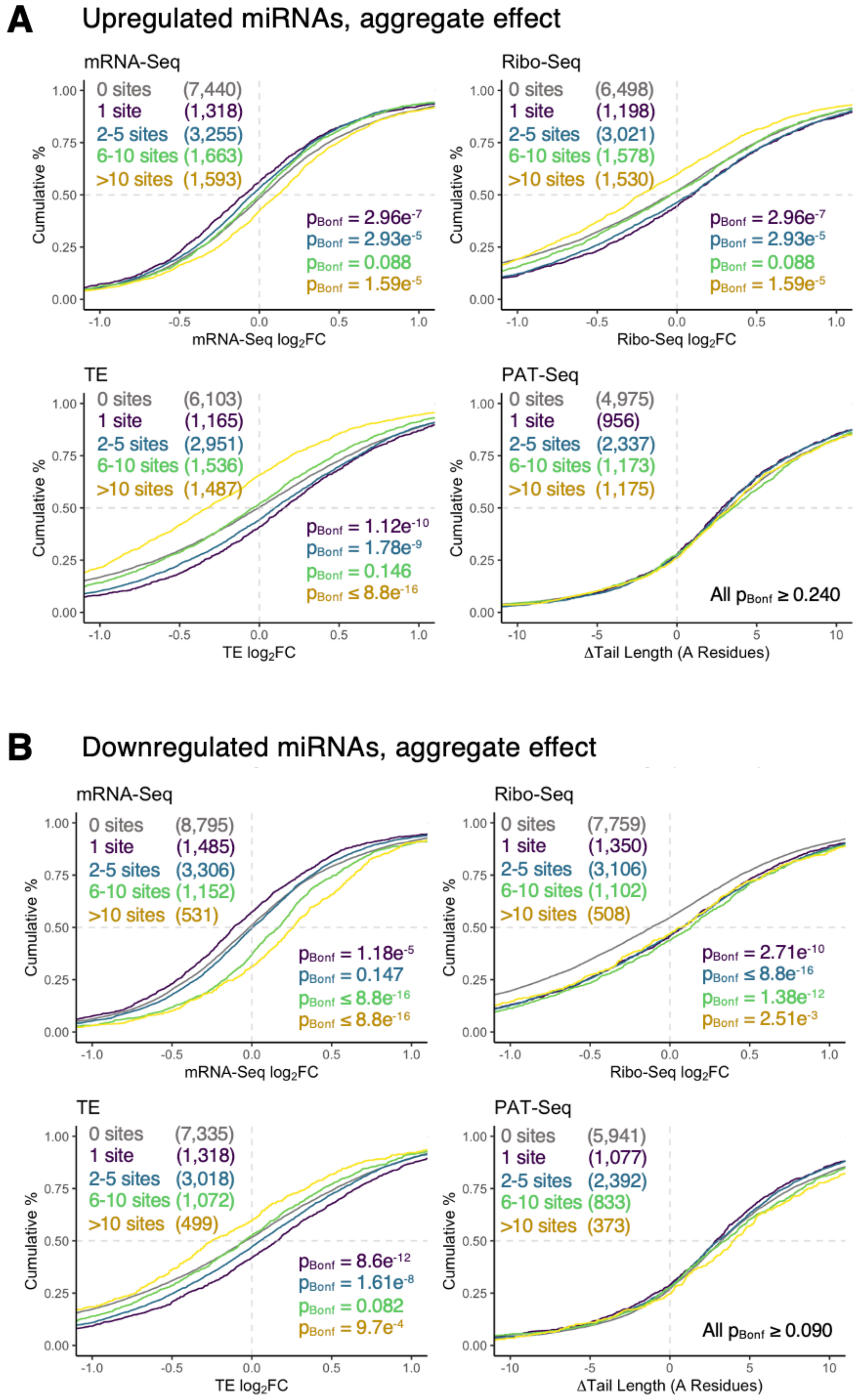

Supplementary Figure 5. Analysis of mRNAs targeted by differentially expressed miRNAs. (A) Cumulative distribution of mRNA expression, translation, translational efficiency and poly(A) tail length for genes targeted by upregulated miRNAs, stratified by the number of predicted binding sites. All groups were compared to genes with no binding sites via Kolmogorov-Smirnov test, with associated Bonferroni-corrected p values reported bottom right. The number of genes per group is reported top left. **(B)** As in **(A)**, except analysing the aggregate effect of downregulated miRNAs.

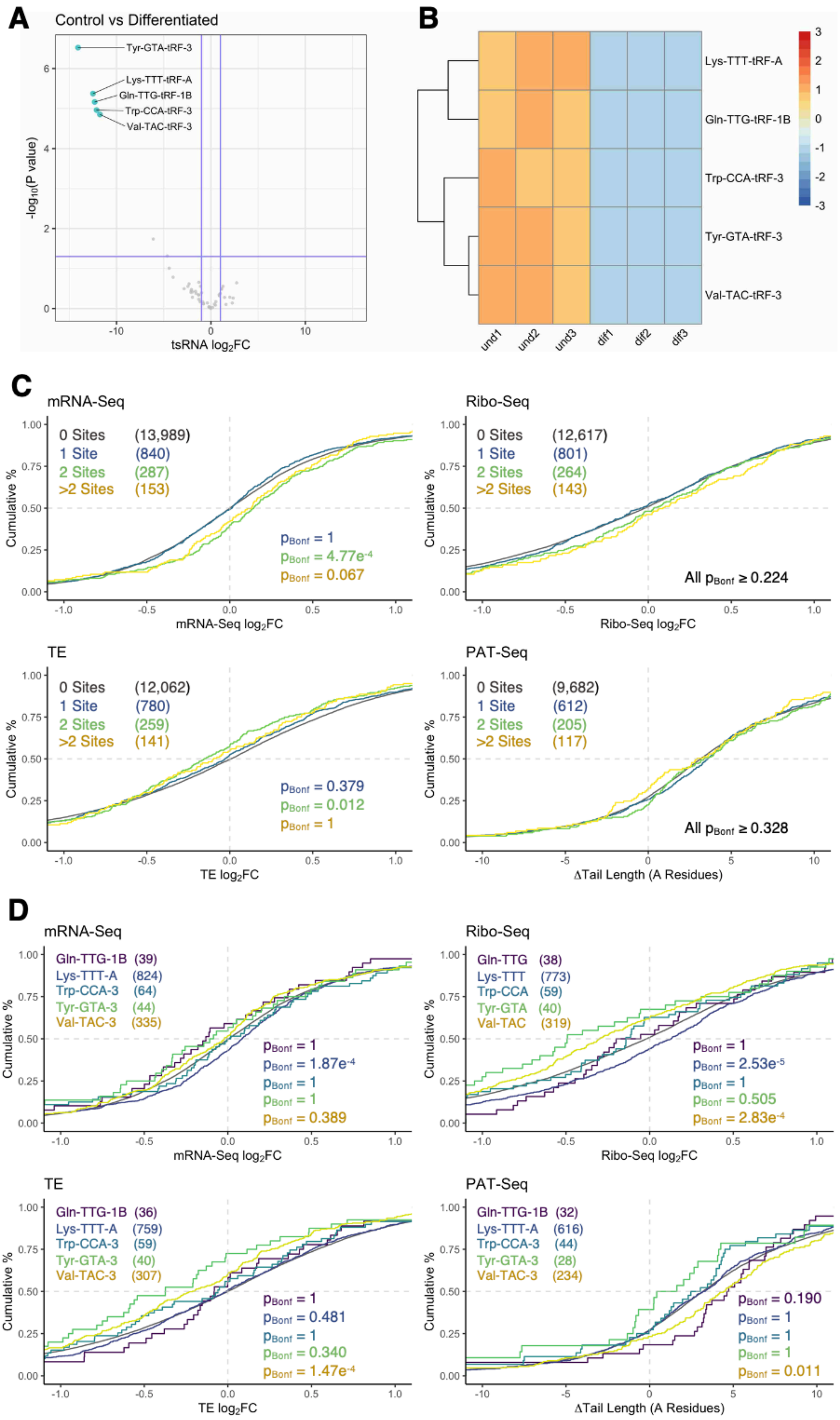
A
Control vs Differentiated
Tyr-GTA-tRF-3
Lys-TTT-tRF-A
Gln-TTG-tRF-1B
Trp-CCA-tRF-3
Val-TAC-tRF-3
-log10(P value)
tsRNA log2FC
B
Lys-TTT-tRF-A
Gln-TTG-tRF-1B
Trp-CCA-tRF-3
Tyr-GTA-tRF-3
Val-TAC-tRF-3
und1
und2
und3
dif1
dif2
dif3
C
mRNA-Seq
0 Sites (13,989)
1 Site (840)
2 Sites (287)
>2 Sites (153)
pBonf = 1
pBonf = 4.77e-4
pBonf = 0.067
Cumulative %
mRNA-Seq log2FC
Ribo-Seq
0 Sites (12,617)
1 Site (801)
2 Sites (264)
>2 Sites (143)
All pBonf ≥ 0.224
Ribo-Seq log2FC
TE
0 Sites (12,062)
1 Site (780)
2 Sites (259)
>2 Sites (141)
pBonf = 0.379
pBonf = 0.012
pBonf = 1
TE log2FC
PAT-Seq
0 Sites (9,682)
1 Site (612)
2 Sites (205)
>2 Sites (117)
All pBonf ≥ 0.328
ΔTail Length (A Residues)
D
mRNA-Seq
Gln-TTG-1B (39)
Lys-TTT-A (824)
Trp-CCA-3 (64)
Tyr-GTA-3 (44)
Val-TAC-3 (335)
pBonf = 1
pBonf = 1.87e-4
pBonf = 1
pBonf = 1
pBonf = 0.389
mRNA-Seq log2FC
Ribo-Seq
Gln-TTG (38)
Lys-TTT (773)
Trp-CCA (59)
Tyr-GTA (40)
Val-TAC (319)
pBonf = 1
pBonf = 2.53e-5
pBonf = 1
pBonf = 0.505
pBonf = 2.83e-4
Ribo-Seq log2FC
TE
Gln-TTG-1B (36)
Lys-TTT-A (759)
Trp-CCA-3 (59)
Tyr-GTA-3 (40)
Val-TAC-3 (307)
pBonf = 1
pBonf = 0.481
pBonf = 1
pBonf = 0.340
pBonf = 1.47e-4
TE log2FC
PAT-Seq
Gln-TTG-1B (32)
Lys-TTT-A (616)
Trp-CCA-3 (44)
Tyr-GTA-3 (28)
Val-TAC-3 (234)
pBonf = 0.190
pBonf = 1
pBonf = 1
pBonf = 1
pBonf = 0.011
ΔTail Length (A Residues)

Supplementary Figure 6. Differential expression of tRNA-derived small RNA fragments and their association with mRNA expression and poly(A) tail length. (A) Volcano plot comparing $\log_2$FC and $-\log_{10}$ p values of tRNA-derived small RNAs (tsRNA) differentially expressed during neuronal differentiation. A total of 5 significantly downregulated tRNA-derived small RNA fragments (tRF; > 14nt and < 28nt) were identified. Suffixes denote location of origin: 1B = body of precursor tRNA, A = parent mature tRNA "A" loop, 3 = parent mature tRNA 3′ end. **(B)** Heat map showing expression profiles of the 5 significantly downregulated tRFs. Each cell corresponds to the sample-level $\log_2$CPM standard deviation relative to the row mean. Red = high expression, blue = low expression. Note that all 5 tRFs were not expressed in differentiated cells, hence the homogeneity amongst these samples. **(C)** Cumulative distribution profiles of mRNA expression, translation, translational efficiency and poly(A) tail length for genes targeted by downregulated tRFs, stratified by the number of predicted binding sites. All groups were compared to genes with no binding sites via Kolmogorov-Smirnov test, with associated Bonferroni-corrected p values reported bottom right. **(D)** As in **(C)**, except examining the target genes of downregulated tRFs individually.

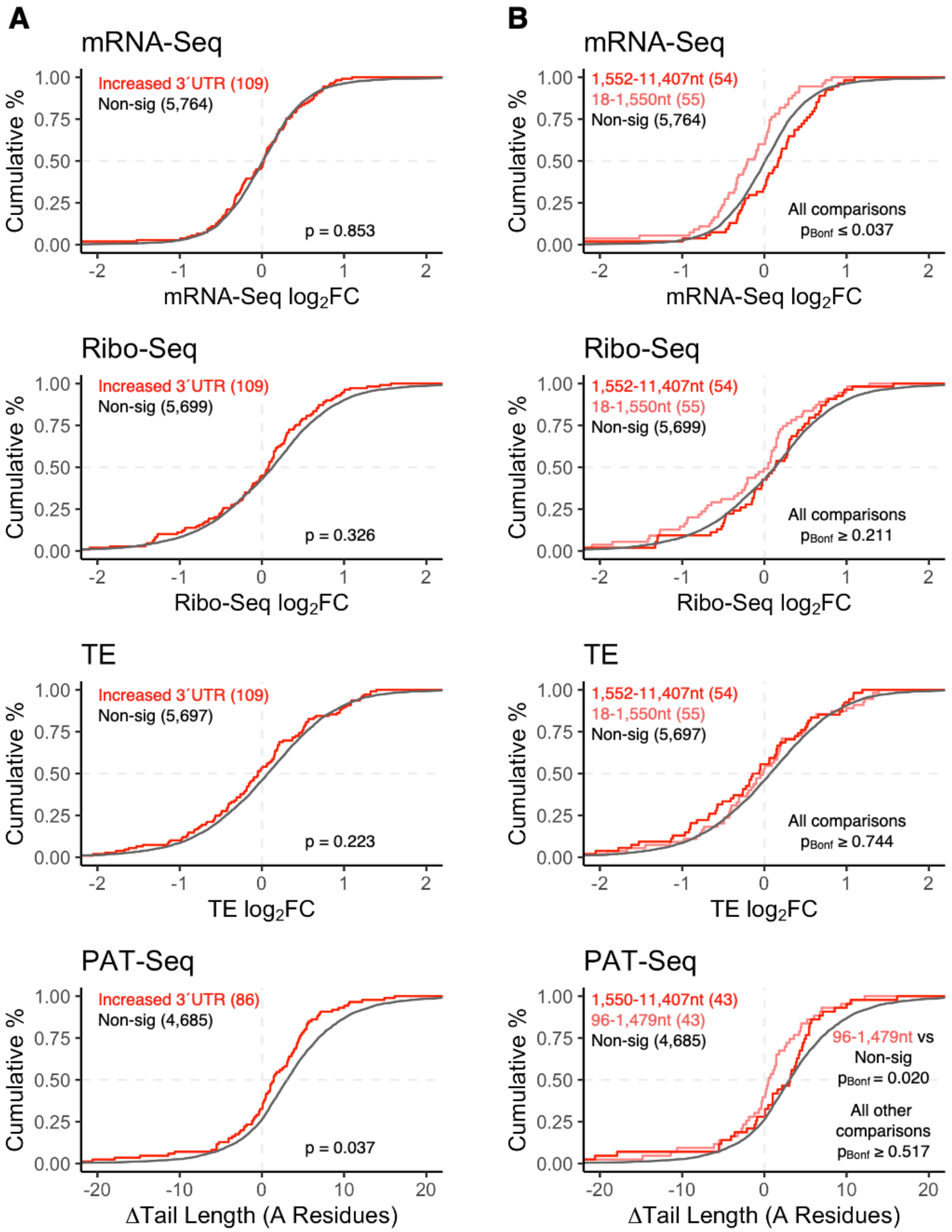

Supplementary Figure 7. Effect of 3´UTR lengthening on mRNA dynamics. (A) Cumulative density plots depicting changes in mRNA expression, translation, translational efficiency and poly(A) tail length for genes with significant 3´UTR lengthening vs genes with no change. Groups were compared via Kolmogorov-Smirnov test, with associated Bonferroni-corrected p values reported bottom right. **(B)** As in **(A)**, with 3´UTR lengthening stratified into halves. All three groups were compared via Kruskal-Wallis test, with post-hoc Dunn's multiple comparisons test conducted to determine pair-wise differences between groups. Bonferroni-corrected p values are reported bottom right.

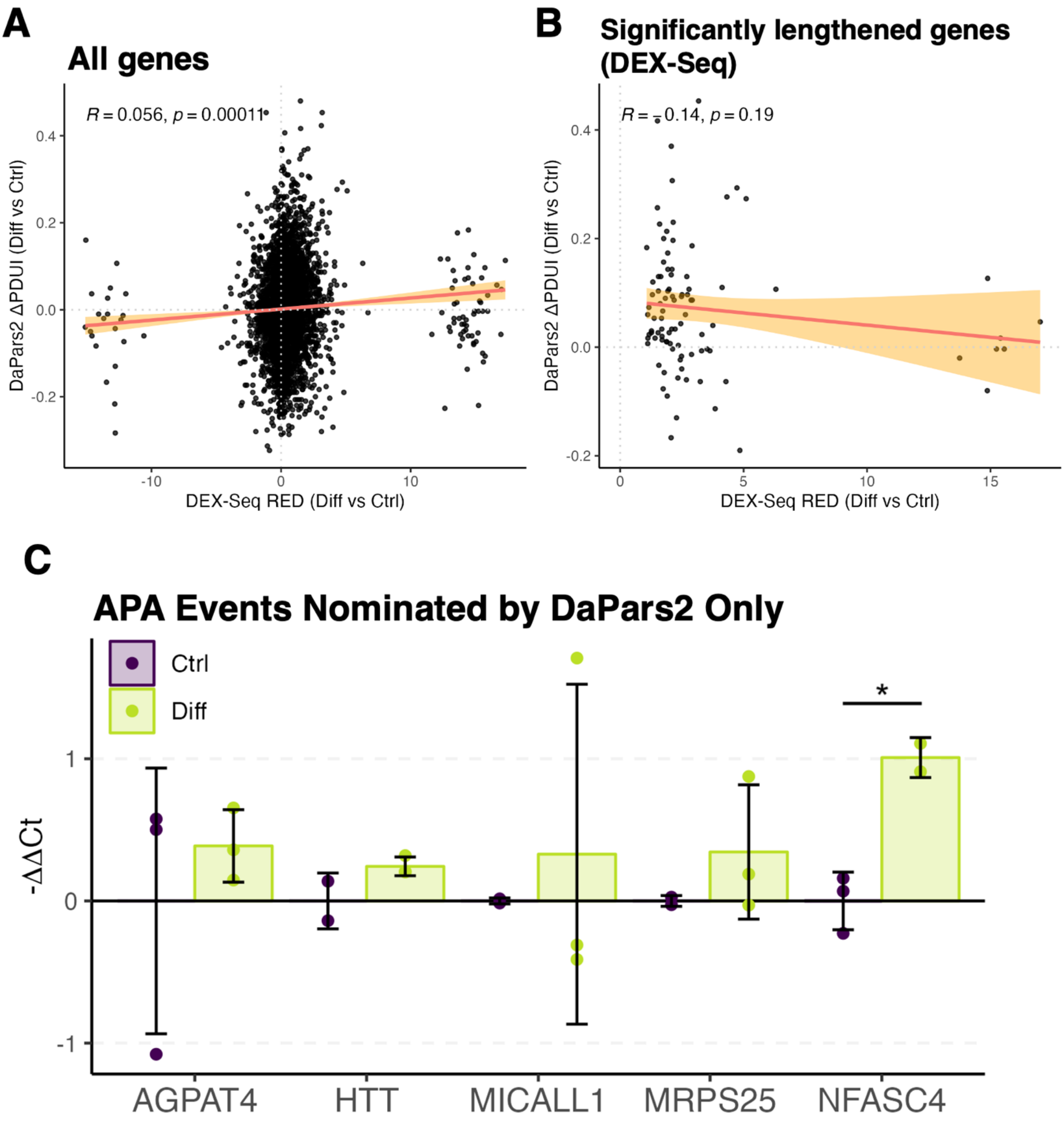

Supplementary Figure 8. Quantification of putative APA events utilising DaPars2 and mRNA-Seq data. (A) Comparison of APA events detected using *DEXSeq* with PAT-Seq data (relative expression difference, RED) and *DaPars2* with mRNA-Seq data (Δ percentage of distal poly(A) site usage, ΔPDUI). Pearson correlation coefficient and associated *P* value reported top left. **(B)** As in **(A)**, except restricted to 110 genes with significant lengthening events as identified via *DEXSeq*. **(C)** qPCR validation of putative APA events detected via *DaPars2* but not *DEXSeq*. Two different primer sets were employed to normalise the expression of alternative 3´UTRs to common upstream regions. ΔCt values between control and ATRA/BDNF differentiated cells were compared via Student's *t*-test, with Benjamini, Kreiger and Yekutieli two-stage linear step-up procedure utilised to correct for multiple testing across all comparisons (* = FDR < 0.05). Data presented as mean –ΔΔCt ± SD.

SUPPLEMENTARY METHODS

mRNA sequencing

mRNA sequencing was conducted using the TruSeq Stranded mRNA Library Preparation Kit, according to the manufacturer's instructions. For each sample, 1µg of high quality (RIN = 8.5) total RNA was diluted to a final volume of 50µL in nuclease-free water and combined with 50µL of oligo-dT RNA purification beads. After gentle mixing, poly(A) RNA was bound to beads via incubation at 65°C for 5 min then room temperature for 5 min. Beads were then concentrated via magnetic stand (5 min), following which supernatant was discarded and unbound RNAs were removed by washing beads with 200µL Bead Washing Buffer (BWB). RNAs bound to oligo-dT beads were then eluted via the addition of 50µL Elution Buffer and incubation at 80°C for 2 min, followed by a 25°C hold. To further purify mRNA from non-specifically bound rRNAs, mRNA was rebound to oligo-dT beads with 50µL Bead Binding Buffer, samples were incubated (room temperature, 5 min), beads were pelleted via magnetic stand and unbound RNA was removed via washing with 200µL BWB.

To fragment and prime the remaining mRNA for reverse transcription, beads were resuspended in 19.5µL Fragment, Prime, Finish Mix and incubated at 94°C for 8 min. Magnetic beads were then pelleted and 17µL of supernatant was collected from each sample for further processing. For first strand synthesis, 8µL of First Strand Synthesis Act D Mix with SuperScript II (1µL SuperScript II per 9µL FSS Act D Mix) was added to each sample and reactions were developed under the following conditions; 25°C for 10 min, 42°C for 15 min, 70°C for 15 min, 4°C hold. Second strand synthesis was immediately conducted by adding 20µL of Second Strand Marking Master Mix followed by incubation at 16°C for 1 hour. Samples were then brought up to room temperature and cDNA was purified using 90µL AMPure XP beads (Beckman Coulter), according to the manufacturer's instructions, with cDNA eluded in 17.5µL Resuspension Buffer (~15µL recovered per sample).

Single adenosine nucleotides were then added to 3′ ends for prior to adapter ligation by adding 12.5µL A-Tailing Mix and incubating samples as per the following program; 37°C for 30 min, 70°C for 5 min, 4°C hold. Adapter ligation was immediately performed by adding 2.5µL Ligation Mix and 2.5µL RNA Adapter Index, with each sample receiving a unique adapter index sequence for downstream library demultiplexing. Reactions were then incubated at 30°C for 10 min and halted via the addition of 5µL Stop Ligation Buffer. Adapter ligated cDNA was then purified via using 42µL AMPure XP Beads, with samples eluted in 52.5µL Resuspension Buffer (~50µL recovered per sample). To ensure successful purification of cDNA, a second purification step was performed using 50µL AMPure XP Beads, with samples eluted in 22.5µL Resuspension Buffer (~20µL recovered per sample).

PCR amplification was next conducted by supplementing samples with 5µL PCR Primer Cocktail and 25µL PCR Master Mix, followed by 15 cycles of PCR as per the following program;

98°C for 10 seconds.
15 cycles of;
 98°C for 10 seconds.
 60°C for 30 seconds.
 72°C for 30 seconds.
72°C for 5 min.

Hold at 4°C.

Amplified cDNA was subsequently purified using 50µL AMPure XP Beads, with samples eluted in 32.5µL Resuspension Buffer (~30µL recovered per sample). Libraries were then validated and quantified by running 1µL of each sample on an Agilent High Sensitivity DNA bioanalyzer chip, after which libraries were normalized to 2nM, pooled (10µL of each library). Prior to sequencing, the combined library was denatured with Sodium hydroxide (200mM) and further diluted with HT1-buffer to a final concentration of 3pM. This diluted library was subjected to 151 single end sequencing cycles using the Illumina NextSeq500 benchtop sequencer.

Ribosome profiling

Ribosome profiling was conducted using the TruSeq Ribo Profile kit (H/M/R; Illumina) as per the manufacturer's instructions, with minor amendments. Translational inhibition was firstly induced by incubating cells (cultured on 10cm plates) in warm culture medium supplemented with 0.1mg/mL cycloheximide (CHX) for 1 min. Cells were then washed with ice-cold PBS supplemented with CHX, after which 800µL of Mammalian Lysis Buffer (Tables M1 & M2) was added to each plate.

Table M1. Mammalian Polysome Buffer Formula

**Reagent**	**Volume (µL)**
500mM Tris*Cl (pH 7.4)	160
5M NaCl	120
1M $MgCl_2$	20
50mg/mL cycloheximide	8
100mM DTT	40
Nuclease-free water	3652
Final volume	4,000

Table M2. Mammalian Lysis Buffer Formula

**Reagent**	**Volume (µL)**
Mammalian Polysome Buffer	887.5
10% Triton X-100	100
2U/µl Turbo DNase I	12.5
Final volume	1,000

Harvest of cells was then executed via extensive scraping on ice, with lysate collected in fresh tubes chilled on ice, shredded by 25-gauge needle, then incubated on ice for 10 min with periodic inversions. Lysates were subsequently clarified by centrifugation (20,000 x g, 4°C, 10 min) and aliquoted into fresh tubes, with a total of ~1mL lysate recovered from each sample.

For ribosome foot printing, 150U RNase I (Invitrogen; optimal concentration determined by RNase titration) was added to 300µL clarified lysate for 45 min at room temperature with gentle mixing, with reactions stopped via addition of 6µL SUPERase In RNase Inhibitor (Invitrogen). Samples were then concentrated to ~100µL via

vacuum centrifugation, after which ribosome protected RNA fragments (RPFs) were purified with MicroSpin S-400 HR columns (GE Healthcare). Columns were first equilibrated with 3mL of 1X Mammalian Polysome Buffer, prior to the addition of 100µL nuclease digested RPF samples and centrifugation (600 x g, room temp, 2 min). The flow-through was then supplemented with 10µL 10% SDS, following which RNA was purified using an RNA Clean & Concentrator-25 kit (Zymo Research), with samples eluted in 26µL nuclease-free water.

Library preparation was then conducted as per the manufacturer's instructions. Ribosomal RNA was firstly depleted from purified RPF RNA using the Ribo-Zero rRNA Removal kit (Illumina), with minor amendments to the manufacturer's protocol. Specifically, the 50°C incubation step during rRNA removal was omitted, while in addition, rRNA-depleted samples were purified via RNA Clean & Concentrator-5 kit (Zymo Research) and eluted in 11µL nuclease-free water. RPFs were then size selected via 12% denaturing urea-polyacrylamide gel electrophoresis (PAGE) as follows. RPF samples were combined with an equal volume of denaturing gel loading dye and denatured at 95°C for 5 min, followed by incubation on ice prior to loading. After pre-running the gel for ~20–30 min, samples were loaded and run at 38W for 90 min to ensure defined band separation. Gels were then stained in darkness with SYBR Gold for 5 min at 4°C, briefly visualised under UV transilluminator and bands ~25–33nt were excised. For purification of RPFs, gel slices were shredded and supplemented with nuclease-free water (400µL), 5M ammonium acetate (40µL) and 10% SDS (2µL). After incubation overnight at 4°C with gentle mixing, gel pieces were removed via 0.22µm Costar Spin-X filter tubes (Corning; 2,000 x g, 3 min) and RPFs were precipitated via the addition of glycogen (4µL) and 100% isopropanol (700µL). After incubation at –20°C for 2 hours, samples were centrifuged (12,000 x g, 4°C, 20 min), RNA pellets were washed with fresh, ice-cold 80% EtOH, then air-dried. Samples were subsequently resuspended in 20µL nuclease-free water.

To prepare RPFs for reverse transcription, end repair was conducted by firstly incubating samples with 7.5µL TruSeq Ribo Profile PNK (Polynucleotide Kinase) buffer (94°C, 25 min), followed by the addition of 3µL TruSeq Ribo Profile PNK and 44.5µL nuclease free water, with samples incubated at 37°C for 1 hour. After clean-up via RNA Clean & Concentrator-5 kit, 1µL TruSeq Ribo Profile 3′ adapter was added and samples were heat denatured (65°C, 2 min). Adapter ligation was then performed by supplementing samples with 6µL ligation master mix (3.5µL TruSeq Ribo Profile Ligation Buffer, 1µL 100mM DTT, 1.5µL TruSeq Ribo Profile Ligase) and incubation at 23°C for 2 hours, followed by the addition of 2µL TruSeq Ribo Profile AR Enzyme and further incubation at 30°C for 2 hours.

Reverse transcription was subsequently performed via the addition of 13µL reverse transcription master mix (Table M3), with samples incubated at 50°C for 30 min. 1µL of TruSeq Ribo Profile Exonuclease was then added to each reaction, after which samples were then incubated with the following program: 37°C for 30 min, 80°C for 15 min, 4°C hold. Samples were then diluted to 50µL total volume with nuclease-free water, after which reactions were cleaned-up via RNA Clean & Concentrator-5 kit. Size selection of cDNA fragments was then performed via 10% native page, with 70-100nt fragments purified as above.

Table M3. Reverse transcription master mix components

**Reagent**	**Volume (µL)**
TruSeq Ribo Profile RT Reaction Mix	4.5

100mM DTT	1.5
Nuclease-free water	6
EpiScript RT	1
Final volume	13

Amplification of cDNA libraries prior to sequencing was achieved utilizing a circularisation PCR method to reduce skewing of libraries towards highly abundant cDNAs, which may result in decreased library complexity. cDNA templates were firstly circularised via the addition of CircLigase master mix (Table M4) and incubation at 60°C for 2 hours. A 7µL aliquot of each circularised sample was then combined with PCR master mix (Table M5), with each sample receiving a unique index PCR primer for downstream library multiplexing. A total of 12 PCR cycles were run as per the following program;

98°C for 30 seconds.

12 cycles of:

94°C for 15 seconds.

55°C for 5 seconds.

65°C for 10 seconds.

Final hold at 4°C.

PCR products were then purified using 90µL Agencourt AMPure XP beads (Beckman Coulter) according to the manufacturer's instructions, after which ~140 – 160bp cDNA libraries were separated from adapter dimers (~113bp) via 8% native PAGE (38W, 70 min, 1% TBE). Libraries were then quantified via qPCR using the KAPA Library Quantification Kit for Illumina Platforms (Roche), normalized to 1nM and pooled (16.67µL of each library). This combined library was subjected to 101 single end sequencing cycles using the Illumina NovaSeq6000 instrument.

Table M4. cDNA master mix components

**Reagent**	**Volume (µL)**
TruSeq Ribo ProfileCL Reaction Mix	4
ATP	2
$MnCl_2$	2
CircLigase	2
Final volume	10

Table M5. PCR master mix components

**Reagent**	**Volume (µL)**
Nuclease-free water	14

TruSeq Ribo Profile Forward PCR Primer	2
TruSeq Ribo Profile Index PCR Primer	2
2x Phusion Master Mix (NEB)	25
Final volume	43

Small RNA sequencing

Preparation of small RNA libraries was conducted via the TruSeq Small RNA Library Prep kit (Illumina), according to the manufacturer's instructions. For each sample, 1µg of high quality (RIN = 10) total RNA was concentrated to 5µL, supplemented with 1µL 3′ adapter and incubated at 70°C for 2 min. Ligation of 3′ adapters was then performed by adding 2µL ligation buffer, 1µL T4 RNA ligase 2 deletion mutant and 1µL RNase inhibitor, followed by incubation at 28°C for 1 hour. Reactions were subsequently halted via the addition of 1µL stop ligation buffer and further incubation at 28°C for 15 min. For 5′ adapter ligation, 1µL of 5′ adapter was incubated at 70°C (2 min) and then supplemented with 1µL ATP and 1µL T4 RNA ligase. This mixture was then added to each sample and reactions were incubated at 28°C for 1 hour.

After adapter ligation, 6µL of adapter-ligated RNA library was combined with 1µL RNA RT primer and incubated at 70°C for 2 min. Reverse transcription was then performed by adding reverse transcription master mix (Table M6) and incubating samples at 50°C for 1 hour. Libraries were then amplified by supplementing samples with Small RNA PCR master mix (Table M7), followed by 11 cycles of PCR as per the following program;

98°C for 30 seconds.
11 cycles of;
    98°C for 10 seconds.
    60°C for 30 seconds.
    72°C for 15 seconds.
72°C for 10 min.
Hold at 4°C.

Each library was then validated and quantified by running 1µL on an Agilent High Sensitivity DNA bioanalyzer chip, after which libraries were normalized to 1nM and pooled (10µL of each library). To purify small RNAs, pooled libraries were subjected to electrophoresis via 6% native polyacrylamide gel, with ~147–157nt bands excised, shredded, supplemented with 200µL nuclease-free water and incubated overnight with gentle agitation to elute cDNA. After removing gel slices as previously described, libraries were precipitated by adding glycogen (2µL), 3M NaOAc (30µL) and 100% EtOH (975µL) followed by centrifugation (17,000 x g, 4°C, 20 min). Supernatant was then discarded, the pellet was washed with 70% EtOH (500µL) and air-dried, after which cDNA was resuspended in 10µL 10mM Tris-HCL, pH 8.5. The pooled library was subsequently quantified via Agilent High Sensitivity DNA bioanalyzer chip, normalized to 2nM and denatured as per the mRNA sequencing protocol. This diluted library was subjected to 76 single end sequencing cycles using the Illumina NextSeq500 benchtop sequencer.

Table M6. Reverse transcription master mix components

Reagent	Volume (µL)
5X First Strand Buffer	2µL
12.5mM dNTP Mix	0.5µL
100mM DTT	1µL
RNase Inhibitor	1µL
SuperScript II Reverse Transcriptase	1µL
Total Volume	5.5µL

Table M7. Small RNA PCR master mix components

Reagent	Volume (µL)
Nuclease-free water	8.5µL
PCR Mix	25µL
RNA PCR Primer	2µL
RNA PCR Primer Index	2µL
Total Volume	37.5µL

Processing and analysis of sequencing data

mRNA sequencing

Raw bcl files obtained via the NextSeq 500 were in Phred33 encoding, stranded and single end. These files were demultiplexed and converted to fastq format via *Bcl2fastq* (version 2.2, Illumina) using the following command;

```
bcl2fastq --sample-sheet <sample.sheet>.csv -o <output.directory>
```

Note that adapter sequences were included in the sample sheet to enable automatic adapter trimming. Output fastq files were subsequently decompressed and lane data were then merged for each sample. Quality control reports were then generated via *FastQC* (version 0.11.5) with the following command;

```
fastqc <input>.fastq
```

The *FastQC* reports were manually inspected for per base quality, as well as overrepresented sequences and adapter content. Reads were then trimmed from the 3′ end based on Phred33 quality score to ensure all 3′ ends were of high quality:

```
cutadapt --nextseq-trim 28 -o <output>.fastq <input>.fastq
```

Alignment to the reference genome (UCSC hg19, obtained from Illumina iGenomes) was then conducted using *HISAT2* (version 2.0.2);

```
hisat2 -x <path/to/hs2.genome.index> -U <input>.fastq -S <output>.sam
```

Finally, reads aligning to features were counted using *HTSeq* (version 0.7.2). Reads were assumed to be stranded and antisense relative to the genome (as indicated by Illumina):

```
htseq-count -f sam -s reverse -t exon <input>.sam
<reference.genome>.gtf > <read.counts>.txt
```

Ribosome profiling

Raw cbcl files obtained via the NovaSeq6000 were in Phred33 encoding, stranded and single end. These files were demultiplexed and converted to fastq format via *Bcl2fastq* (version 2.2, Illumina) using the following command;

```
bcl2fastq --sample-sheet <sample.sheet>.csv -o <output.directory> --
minimum-trimmed-read-length 0
```

Note that the "--minimum-trimmed-read-length 0" flag enables automatic adapter trimming without masking short sequences. Output fastq files were subsequently decompressed and lane data were then merged for each sample. Quality control reports were then generated via *FastQC* (version 0.11.5) with the following command;

```
fastqc <input>.fastq
```

The *FastQC* reports were manually inspected for per base quality, as well as overrepresented sequences and adapter content. Reads between 25–40nt were then selected, after which 3′ bases with Phred33 score < 28 and single 5′ nucleotides were trimmed:

```
cutadapt -m 25 -M 40 -q 28 -u 1 -o <output>.fastq <input>.fastq
```

As Ribo-Seq libraries often exhibit unintended carry-over of small non-coding RNAs, we next aligned processed reads to a fasta reference file (containing rRNA, miRNA, snRNA and snoRNA sequences) with *Bowtie2* (version 2.2.6) and retained unaligned reads for further analysis;

```
bowtie2 -L 20 --un <unaligned.reads>.fastq -x
</path/to/noncoding.RNA.bt2.index> -U <input>.fastq -S
<noncoding.RNA.alignments>.sam
```

Alignment to the reference genome (UCSC hg38, obtained from Illumina iGenomes) was then conducted using *Tophat2* (version 2.2.1), with the output encoded in bam format (default). To ensure that small noncoding RNA reads *not* removed by *Bowtie2* were not aligned, a reference genome with rRNA, miRNA, snRNA and snoRNA loci omitted was used to guide alignment:

```
tophat --output-dir <output.directory> <path/to/bt2.genome.index>
<input>.fastq
```

Finally, reads aligning to features were counted using *HTSeq* (version 0.7.2). Using default parameters, reads were assumed to be stranded and sense relative to the genome (as indicated by Illumina). To ensure that small noncoding RNA reads *not* removed by *Bowtie2* were not counted, a reference genome with rRNA, miRNA, snRNA and snoRNA loci omitted was used to guide read-counting:

```
htseq-count -f <input.file.format> <input>.bam <reference.genome>.gtf
> <read.counts>.txt
```

Small RNA sequencing

Small RNA bcl files were demultiplexed, merged, and subjected to QC as per the mRNA workflow, with the exception that adapters were trimmed using *Cutadapt* to prevent adapter masking during demultiplexing. Reads aligning to tRNA sequences obtained from gtRNAdb (version 2.0) [1] were next identified and filtered from small RNA data using *Bowtie2* (version 2.2.6):

```
bowtie2 -x </path/to/trna.bt2.index> -U <input>.fastq --un
<unaligned.reads>.fastq -S <trna.alignments>.sam
```

Genome alignment was then conducted with *Bowtie2*, with the NCBI GRCh38 reference annotation used to ensure compatibility with the miRBase (version 21) mature miRNA GFF3 annotation;

```
bowtie2 -x </path/to/genome.index> -U <input>.fastq -S
<alignments>.sam
```

Reads aligning to the miRBase mature miRNA annotation file ("hsa.gff3") were then counted with *HTSeq*, with sense strandedness (default) used;

```
htseq-count -f <input>.sam <mature.miRNA.reference>.gff3 >
<read.counts>.txt
```

Analysis of alignment read distribution

The genomic distribution of uniquely aligning reads (i.e. transcription start/stop sites, 5´UTR, CDS, 3´UTR) was conducted with RSeQC (version 2.6.4) as follows;

```
read_distribution.py -i <input>.bam -r hg19_UCSC_knownGene.bed >>
<output>.txt
```

Metagene analysis

The following analyses were performed on mRNA-Seq, Ribo-Seq and PAT-Seq bam files, with all samples merged into a single composite library containing uniquely aligned reads, as these are specifically utilised for read-counting by *HTSeq*. All commands utilised for metagene analyses were sourced from the *Plastid* python library.

Regions of interest around CDS start and stop codons were firstly generated using the *metagene generate* subscript;

```
metagene generate --annotation_files hg19.gtf --landmark cds_stop/cds_start --upstream <number.of.nt.upstream> --downstream <number.of.nt.downstream> <roi.name>
```

For Ribo-Seq libraries, ribosome P-sites were next estimated using the *psite* script and CDS start roi;

```
psite cds.start.roi.txt <output.name> --count_files <input>.bam --min_length 24 --min_counts 1 --normalize_over <number.of.nt.upstream> <number.of.nt.downstream> --require_upstream --constrain 10 15 --aggregate
```

For sub-codon phasing, reads were phased around start codons using the script *phase_by_size.py* as follows. Note that Ribo-Seq reads utilized the variable P-sites as calculated above, whereas offsets were manually set to a fixed distance of 12nt from the 5′ end for mRNA-Seq and PAT-Seq data;

```
# Ribo-Seq

phase_by_size.py cds.start.roi.txt <output.name> --count_files <input>.bam --fiveprime_variable --offset p.sites.txt --min_length 24 --max_length 40

# mRNA/PAT-Seq

phase_by_size.py cds.start.roi.txt <output.name> --count_files <input>.bam --fiveprime --offset 12
```

Metagene profiles around the CDS start and stop codons were then generated with *metagene count*;

```
# Ribo-Seq
```

```
metagene count roi.txt <output.name> --count_files <input>.bam --normalize_over <number.of.nt.upstream> <number.of.nt.downstream> --min_count 1 --use_mean --fiveprime_variable --offset p.sites.txt --min_length 24 --max_length 40

# mRNA/PAT-Seq

metagene count roi.txt <output.name> --count_files <input>.bam --normalize_over <number.of.nt.upstream> <number.of.nt.downstream> --min_count 1 --use_mean --fiveprime --offset 12 --min_length 24 --max_length 151
```

Differential expression analysis

Raw count files (*HTSeq*) from each sample were merged into a single read-count matrix, and reads classed as "no_feature", "ambiguous", "too_low_aQual", "not_aligned" or "alignment_not_unique" were removed. Differential mRNA or RPF expression was then calculated via *EdgeR* (version 3.28.0) as follows. The merged read-count matrix was firstly imported into R and converted into an *EdgeR* DGEList object. A minimum counts-per-million (CPM) threshold was then used to filter out genes with low read counts across all samples. This threshold ensured that genes with a raw read count of 5 in the smallest library were removed. Library normalization factors were then calculated via trimmed mean of means (TMM) method and dispersion was estimated, after which differential expression was determined via exact test. The general analysis pipeline is presented below;

```
# Import edgeR library
library("edgeR")

# Import merged counts
x <- read.delim("merged.counts.txt", row.names="gene_id")

# Assign groups, matching order of columns in merged count file
group <- c("C","C","C","D","D","D")

# Create DGEList object
y <- DGEList(counts=x,group=group)

# Filter out genes with low read-counts across all samples
keep <- rowSums(cpm(y)>1.5) >= 4
y2 <- y[keep,,keep.lib.sizes=FALSE]

# Calculate normalization factors and estimate dispersion
y3 <-calcNormFactors(y2)
y3 <- estimateDisp(y3)

# Determine differential expression via exact test
et <- exactTest(y3, c("C", "D"))
```

Differential translational efficiency

Analysis of differential translational efficiency was conducted via the *RiboDiff* (version 0.2.1) package. Raw mRNA and RPF counts were firstly merged into a single matrix, after which genes surviving *edgeR* CPM thresholds were selected for further analysis. A csv file containing sample names, data types (mRNA-Seq/Ribo-Seq) and experimental conditions was additionally produced to guide translational efficiency analysis. Translational efficiency was then calculated using the following script, with a *P* value threshold set to 1 to ensure all results were printed;

```
python ribodiff/scripts/TE.py -p 1 -e <phenotype.file>.csv -c
<merged.counts>.txt -o <output>.txt
```

Identification of tsRNAs and target gene prediction

Small RNA reads aligning to tRNAs were analysed for tRNA-derived small RNAs (tsRNA) using the *tDR Mapper* package [1]. Reads were firstly aligned to mature and precursor human tRNA sequences sourced from gtRNAdb (v2.0) [2], progressively allowing for exact matches, then one, two or three mismatches or deletions. Consecutive positions of >50% coverage were then used to determine the primary tsRNA associated with the parent tRNA, with regions > 14nt and < 28nt defined as tRNA fragments (tRF) and regions ≥ 28nt and < 41nt defined as tRNA halves (tRH). For each sample, tsRNA read-counts were then assembled. If a tsRNA was derived from multiple parent tsRNAs with the same anticodon and amino acid, the average of all read-counts was taken to mitigate the inclusion of identical tsRNA sequences in downstream analyses. Differential tsRNA expression was then calculated using *edgeR* as described above.

To investigate the potential for tRFs to regulate mRNA expression in a similar manner to miRNAs, TargetScan custom prediction scripts (perl) [3] were used to identify potential tRF-mRNA interactions for further analysis. For this analysis, nucleotides 2-8 were specified as the tRF “seed region”. For each gene, the context scores of all potential binding sites were aggregated to produce total context+ scores, as previously described [4]. Predicted tRF-gene pairings with total context+ score < –0.2 were then analysed for changes in mRNA expression and poly(A) tail length as per the miRNA analysis.

RT-qPCR

To confirm mRNA, miRNA and APA differential expression, 1µg of total RNA was reverse transcribed per sample for production of both miRNA and mRNA cDNA. For miRNA, total RNA was polyadenylated with 1.25 units (U) of *E. coli* poly(A) polymerase (NEB) and 1mM ATP. For both mRNA and miRNA, genomic DNA was depleted with 1U amplification-grade DNase I at room temperature for 13 min, with reactions blocked using 25mM EDTA at 65°C for 10 min. Reverse transcription was then performed using 200U SuperScript II reverse transcriptase (Invitrogen) as per the manufacturer’s instructions, with 40U RNaseOUT RNase inhibitor (Invitrogen) included to limit RNA degradation. Reactions were primed with either 5ng/µL random hexamers (mRNA) or 2µM miRNA universal primers (5′-CAGGTCCAGTTTTTTTTTTTTTTTVN-3′, where V = A, G or C and N = A, T, G or C) [5] for enhanced miRNA specificity. Negative control reactions were included by substituting SuperScript II with nuclease-free water.

Quantitative real-time PCR (qPCR) was conducted with 5µL of a 1/20 cDNA dilution, 0.5µL of both forward and reverse primers (10µM, see Table S1 for primer sequences and supplementary methods for design considerations),

6.25µL Power SYBR Green master mix (Applied Biosystems, Foster City, CA, USA) and 0.25µL nuclease-free water. Reactions were run on an Applied Biosystems 7500 Real Time PCR System, with a dissociation curve to assess primer specificity. For analysis of mRNA expression, the geometric mean of *GUSB* (β-glucuronidase) and *TUBB2A* (tubulin β-2A) was used as a reference, which was subtracted from the target mRNA cycle threshold (C_t) to determine ΔC_t. For miRNAs, *U6* and *U49* small nucleolar RNAs were instead employed as a reference. For APA, primers were designed to amplify either the entire gene, or only the long 3´UTR isoform (as identified via the APA analysis). The C_t of the target gene was then subtracted from C_t of the long APA isoform to identify changes in APA site preference, corrected for overall changes in gene expression. Statistical analysis of ΔC_t changes between conditions was determined via two-sided Student's t-test, with correction for multiple testing via Benjamini, Krieger and Yekutieli two-stage linear step-up procedure. An FDR < 0.05 was considered statistically significant.

Primer design

All miRNA primers were designed using the *miRprimer* software package [6], which generates primers compatible with miRNA cDNA produced via the methodology described above. Primer design for the APA qPCRs was conducted via the NCBI Primer Blast tool [7] and the Integrated DNA Technologies (IDT) OligoAnalyzer Tool. Primers were designed to comply with the following criteria:

1. Melting temperature (T_m) near 60°C and < 3° difference between forward and reverse primers.
2. GC% between 40 - 60%, with the exception of miRNA reverse primers.
3. No secondary structures with free energy (ΔG) < -2 kcal/mol and/or within 15°C of the T_m.
4. No homodimers or heterodimers with 3´ complementarity.
5. No 5´ or internal homodimers or heterodimers with ΔG < -8 kcal/mol.

Identification of APA events from the mRNA-Seq data

APA events were identified from the mRNA-Seq data using *DaPars2* [8]. Putative APA events supported by a minimum 10 counts were identified from mRNA-Seq bamfiles using the *Dapars2_Multi_Sample.py* command, in conjunction with the *hg19_refseq_extracted_3UTR.bed* 3´UTR reference (available from https://github.com/ZhengXia/dapars/blob/master/DaPars_Test_Dataset.zip). For each gene, utilisation of distal poly(A) sites was quantified as a percentage of distal poly(A) site usage index (PDUI), noting that we retained the most highly expressed site per gene. Changes in PDUI (ΔPDUI) between control and differentiated cells were then analysed via *t*-test, with *P* values adjusted via Benjamini-Hochberg *FDR*. We note that sites with no change between control and differentiated cells were omitted, as well as sites with a standard deviation of zero in both conditions, since these sites produce infinite *t*-statistics. To compare results between the PAT-Seq and mRNA-Seq APA analyses, Pearson correlation was analysed between *DEXSeq* RED values and *DaPars2* ΔPDUI values for genes detected in both datasets.

REFERENCES

1. Selitsky, S.R. and P. Sethupathy, *tDRmapper: challenges and solutions to mapping, naming, and quantifying tRNA-derived RNAs from human small RNA-sequencing data.* BMC Bioinformatics, 2015. **16**: p. 354.
2. Chan, P.P. and T.M. Lowe, *GtRNAdb 2.0: an expanded database of transfer RNA genes identified in complete and draft genomes.* Nucleic Acids Res, 2016. **44**(D1): p. D184-9.
3. Agarwal, V., et al., *Predicting effective microRNA target sites in mammalian mRNAs.* Elife, 2015. **4**.
4. Garcia, D.M., et al., *Weak seed-pairing stability and high target-site abundance decrease the proficiency of lsy-6 and other microRNAs.* Nat Struct Mol Biol, 2011. **18**(10): p. 1139-46.
5. Balcells, I., S. Cirera, and P.K. Busk, *Specific and sensitive quantitative RT-PCR of miRNAs with DNA primers.* BMC Biotechnol, 2011. **11**: p. 70.
6. Busk, P.K., *A tool for design of primers for microRNA-specific quantitative RT-qPCR.* BMC Bioinformatics, 2014. **15**: p. 29.
7. Ye, J., et al., *Primer-BLAST: a tool to design target-specific primers for polymerase chain reaction.* BMC Bioinformatics, 2012. **13**: p. 134.
8. Xia, Z., et al., *Dynamic analyses of alternative polyadenylation from RNA-seq reveal a 3'-UTR landscape across seven tumour types.* Nat Commun, 2014. **5**: p. 5274.
